# A self-amplified nanocatalytic system for achieving “1 + 1 + 1 > 3” chemodynamic therapy on triple negative breast cancer

**DOI:** 10.1186/s12951-021-00998-y

**Published:** 2021-09-04

**Authors:** Lulu Zhou, Jinjin Chen, Yunhao Sun, Keke Chai, Zhounan Zhu, Chunhui Wang, Mengyao Chen, Wenmei Han, Xiaochun Hu, Ruihao Li, Tianming Yao, Hui Li, Chunyan Dong, Shuo Shi

**Affiliations:** 1 Shanghai Key Laboratory of Chemical Assessment and Sustainability, School of Chemical Science and Engineering, Breast Cancer Center, Shanghai East Hospital, Tongji University, Shanghai, 200092 People’s Republic of China; 2 Department of Oncology, The Fourth Affiliated Hospital of Nantong University, First People’s Hospital of Yancheng, Yancheng, Jiangsu People’s Republic of China; 3 Department of Thoracic Surgery, First People’s Hospital of Yancheng, Affiliated to Medical College of Nanjing University, Yancheng, Jiangsu People’s Republic of China

## Abstract

**Background:**

Chemodynamic therapy (CDT), employing Fenton or Fenton-like catalysts to convert hydrogen peroxide (H_2_O_2_) into toxic hydroxyl radicals (·OH) to kill cancer cells, holds great promise in tumor therapy due to its high selectivity. However, the therapeutic effect is significantly limited by insufficient intracellular H_2_O_2_ level in tumor cells. Fortunately, β-Lapachone (Lapa) that can exert H_2_O_2_-supplementing functionality under the catalysis of nicotinamide adenine dinucleotide (phosphate) NAD(P)H: quinone oxidoreductase-1 (NQO1) enzyme offers a new idea to solve this problem. However, extensive DNA damage caused by high levels of reactive oxygen species can trigger the “hyperactivation” of poly(ADP-ribose) polymerase (PARP), which results in the severe interruption of H_2_O_2_ supply and further the reduced efficacy of CDT. Herein, we report a self-amplified nanocatalytic system (ZIF67/Ola/Lapa) to co-deliver the PARP inhibitor Olaparib (Ola) and NQO1-bioactivatable drug Lapa for sustainable H_2_O_2_ production and augmented CDT (“1 + 1 + 1 > 3”).

**Results:**

The effective inhibition of PARP by Ola can synergize Lapa to enhance H_2_O_2_ formation due to the continuous NQO1 redox cycling. In turn, the high levels of H_2_O_2_ further react with Co^2+^ to produce the highly toxic ·OH by Fenton-like reaction, dramatically improving CDT. Both in vitro and in vivo studies demonstrate the excellent antitumor activity of ZIF67/Ola/Lapa in NQO1 overexpressed MDA-MB-231 tumor cells. Importantly, the nanocomposite presents minimal systemic toxicity in normal tissues due to the low NQO1 expression.

**Conclusions:**

This design of nanocatalytic system offers a new paradigm for combing PARP inhibitor, NQO1-bioactivatable drug and Fenton-reagents to obtain sustained H_2_O_2_ generation for tumor-specific self-amplified CDT.

**Graphic Abstract:**

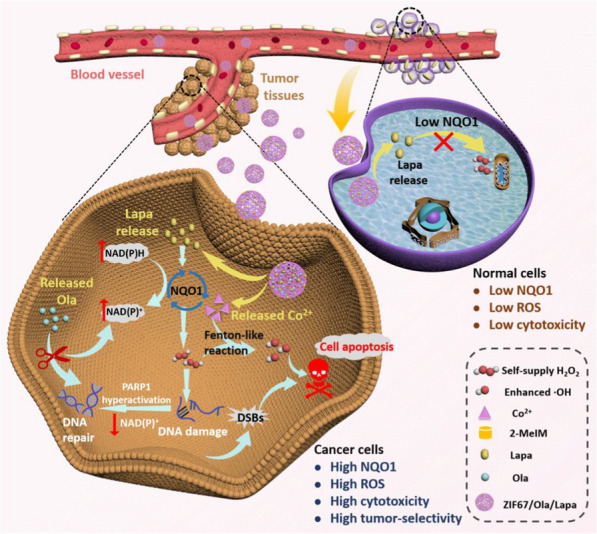

**Supplementary Information:**

The online version contains supplementary material available at 10.1186/s12951-021-00998-y.

## Introduction

As a newly developed reactive oxygen species (ROS)-mediated therapeutic model, chemodynamic therapy (CDT) that can transform intratumoral overexpressed hydrogen peroxide (H_2_O_2_) into highly cytotoxic ·OH with the participation of Fenton or Fenton-like regents, such as Fe^2+^, Fe^3+^, Mn^2+^, Cu^+^ and Co^2+^ has aroused great attention [1–7]. Compared to photodynamic or sonodynamic therapy, CDT can be performed independently without external energy input (light or ultrasonic) and oxygen, which enables it to circumvent the drawbacks of limited tissue penetration depth, hypoxic tumor microenvironment and nonspecificity [8–13]. To date, various elaborately designed Fenton nanocatalysts have been exploited to catalyze the conversion of endogenous H_2_O_2_ in tumor cells into ·OH for cancer CDT [14–17]. Nevertheless, the insufficient endogenous concentration of H_2_O_2_ in cancer cells undoubtedly confers a large chemical barrier for effective CDT [18–21]. In order to cope with the lack of H_2_O_2_ in tumors, multiple efforts have been devoted to increasing the intratumoral H_2_O_2_ concentration for CDT-efficacy enhancement, including encapsulating exogenous H_2_O_2_ [3, 22] or facilitating the H_2_O_2_ generation in tumor regions [9, 23, 24]. However, it is difficult to completely deliver the wrapped H_2_O_2_ to the tumor site, and the leakage of H_2_O_2_ from nanocarriers can induce non-specific toxicity. In this regard, in situ production of H_2_O_2_-supplementing functionality into CDT agents may be a better choice to improve the anticancer efficacy.

β-Lapachone (Lapa), a unique NQO1 bioactivatable drug, is capable of producing massive H_2_O_2_ under the catalysis of nicotinamide adenine dinucleotide (phosphate) NAD(P)H: quinone oxidoreductase-1 (NQO1) enzyme [25, 26]. By utilizing NAD(P)H as an electron donor, NQO1 triggers the futile redox cycling between the quinone and hydroquinone forms of Lapa, in which 60 mol of NAD(P)H are consumed to create 120 mol of H_2_O_2_ in 2 min [27, 28]. More importantly, since the NAD(P)H: NQO1 is constitutively overexpressed at levels 5- to 200-fold greater in breast [29], non-small cell lung [28], pancreas [27, 30], and prostate cancer [31] compared with the associated normal tissues, selecting Lapa for tumor-specific H_2_O_2_ level amplification seems to hold great promise for enhanced CDT efficacy.

It is noted that tumor cells with elevated intrinsic H_2_O_2_ stress are more vulnerable to cause DNA lesions, and when DNA damages overwhelm repair capacity, poly(ADP-ribose) polymerase (PARP) becomes hyperactivated [28]. PARP is essential for DNA base excision repair, single-strand break (SSB), and double-strand break (DSB) repair [26, 28]. The hyperactivation of PARP is accompanied by severe NAD^+^ pool (its substrate) and ATP loss, exhaustion of NQO1 futile redox [26, 29], and further interruption of H_2_O_2_ supply. Therefore, the synergy of PARP inhibitor (PARPi) and Lapa is a promising strategy to extend NQO1-mediated H_2_O_2_ generation, inhibit PARP-driven DNA repair and enhance DNA damages in a tumor-selective manner. In the previous clinical study, Olaparib (Ola)-induced inhibition of PARP has demonstrated success in patients with BRCA-mutated advanced ovarian cancer and breast cancer [32]. Taking all the above into consideration, it is expected that the combination modality of Lapa and Ola will serve as a simple and promising H_2_O_2_ supplier to augment CDT efficacy. To the best of our knowledge, there is no report on the combination of Lapa, Ola and Fenton-reagents to achieve self-amplified CDT.

**Scheme 1:**
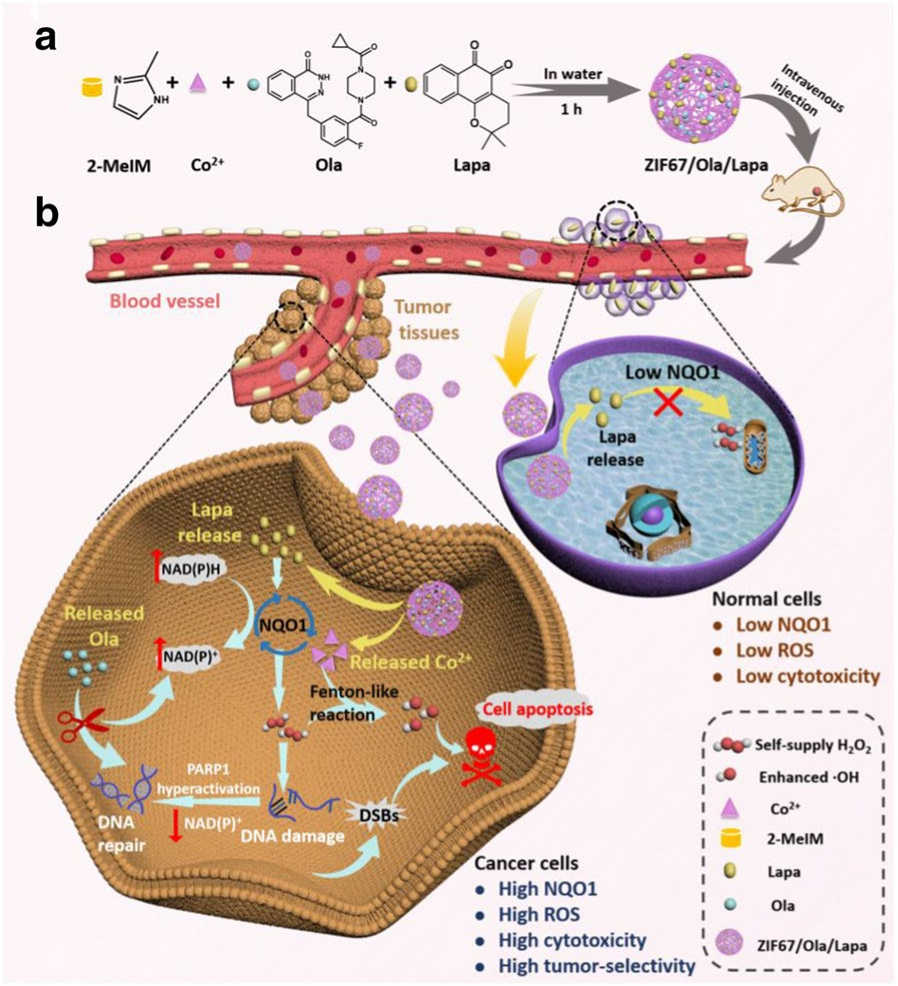
**a** Formation of the ZIF67/Ola/Lapa nanoparticles. **b** The mechanism of collaborative enhancement of CDT efficacy based on sustained NQO1-mediated H_2_O_2_ production caused by the synergy between Lapa and Ola (PARPi)

Herein, we present a nanocatalytic system that possesses amplified H_2_O_2_ self-supplying property for tumor-specific growth inhibition by enhanced CDT (Scheme 1). The nanoplatform was constructed by a facile one-pot process using cobalt-based metal–organic framework (ZIF67) as drug carrier to co-encapsulate anticancer drug Lapa and PARPi Ola. Such a nanocatalytic system was designed based on the following considerations: (1) the zeolitic imidazolate framework ZIF67 is chosen as the drug delivery system by virtue of its high loading capacity, excellent biocompatibility and good biodegradability [33]. Under acid environment, ZIF67 will disintegrate, and Co^2+^ as the degradation product can catalyze H_2_O_2_ to produce highly toxic ·OH through a Fenton-like reaction. (2) Due to the overexpression of NQO1 in tumor cells, the ZIF67/Ola/Lapa nanoparticles are selectively enriched in tumor tissues. Subsequently, ZIF67 will be disassembled in the acidic environment of tumor due to its pH sensitivity, resulting in the release of Co^2+^, Ola and Lapa. (3) On the one hand, the released Lapa undergoes a futile redox cycle resulting in the rapid production of H_2_O_2_, specifically catalyzed by NQO1 enzyme. High levels of H_2_O_2_ can induce massive oxidative bases and DNA lesions, which overwhelms DNA repair capacity and causes “hyperactivation” of PARP, accompanied by a dramatic NAD^+^/ATP depletion and further exhaustion of NQO1 futile redox. On the other hand, the released Ola, as the PARP inhibitor, can block PARP-driven DNA repair, prevent NAD^+^ loss, sustain NQO1 futile cycling, and cause elevated H_2_O_2_ levels. High levels of H_2_O_2_ further react with Co^2+^ to produce highly toxic ·OH through a Fenton-like reaction, and eventually induce apoptosis. Owing to the low NQO1 expression in normal cells, the ZIF67/Ola/Lapa nanoparticles will not cause significant H_2_O_2_ level amplification and severe side effects in normal tissues. Therefore, this nanocatalytic system is a promising strategy for tumor-specific enhanced chemodynamic therapy.

## Materials and methods

### Synthesis of ZIF67/Ola/Lapa nanoparticles

Briefly, 16 mg of cobaltous nitrate hexahydrate was dissolved in 2 mL of deionized water. Then, 2 mL of 2-methylimidazole (259.6 mg) mixed with 1 mg Ola and 2 mg Lapa was added to the as-prepared cobaltous solution. After being stirred for 1 h, the product was collected by centrifugal separation (12,000 rpm, 5 min). The obtained precipitates were washed three more times with deionized water. Finally, purplish red Ola/Lapa-encapsulated ZIF67 nanoparticles were dried at 40 °C under vacuum. The pure ZIF67, ZIF67/Ola and ZIF67/Lapa nanoparticles were also prepared by a similar procedure.

### ·OH generation by ZIF67/Ola/Lapa mediated Fenton-Like reaction

In the 25 mM NaHCO_3_ buffer solution (2 mL), MB solution (1 mg/mL, 20 μL), H_2_O_2_ (1 M, 20 μL) and ZIF67/Ola/Lapa solution (the final concentration was 100 μg/mL) were mixed together. After incubating at 37 °C for 2 h, the solutions were centrifuged and the absorbance was measured by UV–Vis spectroscopy [34].

### In vitro cytotoxicity assay

For cell viability measurements, the cells were plated in 96-well at a density of 10 × 10^3^ cells per well. Following incubation for 24 h, samples were added in fresh DMEM. Samples consisting of ZIF67, Ola, Lapa, ZIF67/Ola, ZIF67/Lapa, Ola + Lapa and ZIF67/Ola/Lapa. MDA-MB-231 cells were exposed to different concentrations of samples for 24 h. 100 μL of DMEM (without FBS) containing 10% CCK8 was added into each well. After 4 h incubation at 37 °C, the absorbance at 450 nm was measured using a standard enzyme linked immunosorbent assay (ELISA) format spectrophotometer. Experiment was repeated three times and the cell viability was calculated using the following formula:

$$\begin{aligned} {\text{Cell viability}}{\mkern 1mu} = {\mkern 1mu} & \left( \begin{gathered} {\text{mean absorbance of test wells}}{\mkern 1mu} \hfill \\ - {\mkern 1mu} {\text{mean absorbance of medium control wells}} \hfill \\ \end{gathered} \right) \\ & /\left( \begin{gathered} {\text{mean absorbance of untreated wells}}{\mkern 1mu} \hfill \\ - {\mkern 1mu} {\text{mean absorbance of medium control well}} \hfill \\ \end{gathered} \right){\mkern 1mu} \times {\mkern 1mu} 100\% . \\ \end{aligned}$$

### Determination of intracellular ATP, NAD^+^ and NADH level

For ATP assay, the supernatant of cell culture was taken by double antibody one-step sandwich enzyme linked immunosorbent assay (ELISA) (ATP ELISA Kit instruction). The samples, standard samples and HRP-labeled detection antibodies were added into the micropores coated with ATP-trapping antibodies. The samples were incubated and washed thoroughly. The substrate TMB is used to produce color, which is converted to blue by peroxidase and to the final yellow by acid. The depth of the color is positively correlated with the ATP in the sample. The absorbance (OD value) was measured at 450 nm with a microplate analyzer, and the concentration of the sample was calculated. For NAD^+^ and NADH measurements, NAD^+^ and NADH were extracted and quantified from cell lysates using a specific kit (Abcam, ab65348) following the manufacturer’s instructions that exclude interaction with NADP and NADPH.

### Western blot assay

MDA-MB-231 cells were collected after different treatments and total cellular protein was extracted. The concentration of protein was measured by a bicinchoninic acid protein assay kit (Catalogue no. 23225, Thermo). Then 50 μg of proteins were used for SDS-PAGE electrophoresis and further transferred to 0.22 μm PVDF membranes. After incubated with Caspase-3 antibody (Catalogue no. 10442-1-AP, Proteintech), Bcl-2 antibody (Catalogue no. 12789-1-AP, Proteintech), and BAX antibody (Catalogue no. 13371-1-AP, Proteintech) at 4 °C overnight, these membranes were washed for 3 times and incubated with secondary antibodies at RT for 1 h. After washed for 3 times, the immunoreactivity was visualized after incubating with enhanced chemiluminescence (ECL) reagents. GAPDH was chosen as a loading control.

### In vivo biodistribution of ZIF67/Ola/Lapa nanoparticles

When the tumors grew to 200–300 mm^3^ in volume, MDA-MB-231 tumor-bearing mice were iv administrated with ICG-ZIF67/Ola and ICG-ZIF67/Ola/Lapa (ICG, 0.5 mg/kg). Thereafter, the mice were anesthetized and imaged in a small animal imaging system (Anyview 100, Biolight Biotechnology, Guangzhou) at 1, 2, 6, 12, 24 h post-injection. The excitation wavelength was 740 nm and the fluorescence emission at 820 nm was collected. For the tissue distribution study of the composites, the mice were sacrificed at 12 h post-injection, and major organs were collected for ex vivo imaging.

### In vivo anti-tumor therapy

MDA-MB-231 tumors bearing mice were randomly divided into seven groups (five mice per group) for the treatments with PBS, Ola, Lapa, Ola + Lapa, ZIF67/Ola, ZIF67/Lapa and ZIF67/Ola/Lapa. Treatments were started (designated as day 0) when MDA-MB-231 tumors reached a volume of 100–150 mm^3^. Distinct formulations were administered via tail vein injections at multiple doses (an equivalent dose of 2.4 mg Lapa per kg) on every other day for six times. Mouse weights and tumor sizes were measured every other day. Tumor size was recorded by a caliper and tumor volume was defined as:

$${\text{V}}\, = \,{\text{L}}\, \times \,{\text{W}}^{{2}} /{2},$$

where L and W were the abbreviation of tumor length and width, respectively. The tumor growth was observed after drug withdrawal continuously, and the rats were sacrificed on the 14th day and then the tumors were excised and weighed. Simultaneously, the main organs (heart, liver, spleen, lung, and kidney) and tumor were obtained and utilized for H&E analysis.

### Immunohistochemistry and TUNEL

The tumor was fixed with 4% paraformaldehyde and embedded in paraffin, which was further cut into 5 μm sections for immunohistochemical staining of Caspase-3 (1:150; GB11009-1; Servicebio), Bcl-2 (1:100; GB13439; Servicebio), and γ-H2AX (1:150; GB111841; Servicebio). To evaluate cellular apoptosis, the tumor section was stained using aterminal deoxynucleotidyl transferase-mediated deoxyuridine triphosphate nick end labeling (TUNEL) assay kit (KeyGEN BioTECH, Nanjing) according to the manufacturer’s protocols.

### Hematology examination

The murine blood was acquired from each group (n = 3) at the end of the experiment. After centrifugation at 4 °C for 20 min, the plasma was collected for blood biochemical analysis. The concentration of alanine amino transferase (ALT) was determined to evaluate liver function. The concentration of blood creatinine (CREA) levels was determined to evaluate renal function. The whole blood was also collected for white blood cells and hemoglobin analysis.

### ROS production in tumor tissues

The production of ROS in tumors was detected via a fluorescence probe DHE (Catalogue no. D7008, Servicebio). When the tumors grew to 200–300 mm^3^ in volume, distinct formulations were administered via tail vein injections at multiple doses (an equivalent dose of 2.4 mg Lapa per kg). After 6 h injection, the mice were sacrificed, and the tumors were excised and sectioned into 10 μm thick slices, which were incubated with DHE (1:400) diluted with PBS at 37 °C for 30 min under dark conditions. Then the slices were dried slightly and DAPI dye solution was added to stain the nuclei at room temperature for 10 min. The slides were then washed with PBS (pH 7.4) for 3 times. After slightly drying, the slices were sealed with anti-fluorescence quenching sealing tablet. The slices were observed for CLSM observation and the images were collected (DAPI: Ex: 330–380 nm, Em: 420 nm; FITC: Ex: 450 nm, Em: 520 nm). The integrated optical density of fluorescence was analyzed with Image J software. The average fluorescence intensity in each mouse was calculated on the basis of 5 images. And the fluorescence intensities and standard errors were determined from 3 mice.

### Statistical analysis

Representative results were obtained from at least three independent experiments. All data were statistically analyzed by SPSS 21.0 software. The counting data were expressed as percentage, and χ^2^ test was used between groups. Measurement data were expressed as mean ± standard deviation, and mean comparison between groups was tested by one-way ANOVA. P < 0.05 was considered statistically significant.

## Results and discussion

### Preparation and characterizations of the ZIF67/Ola/Lapa nanoparticles

**Fig. 1:**
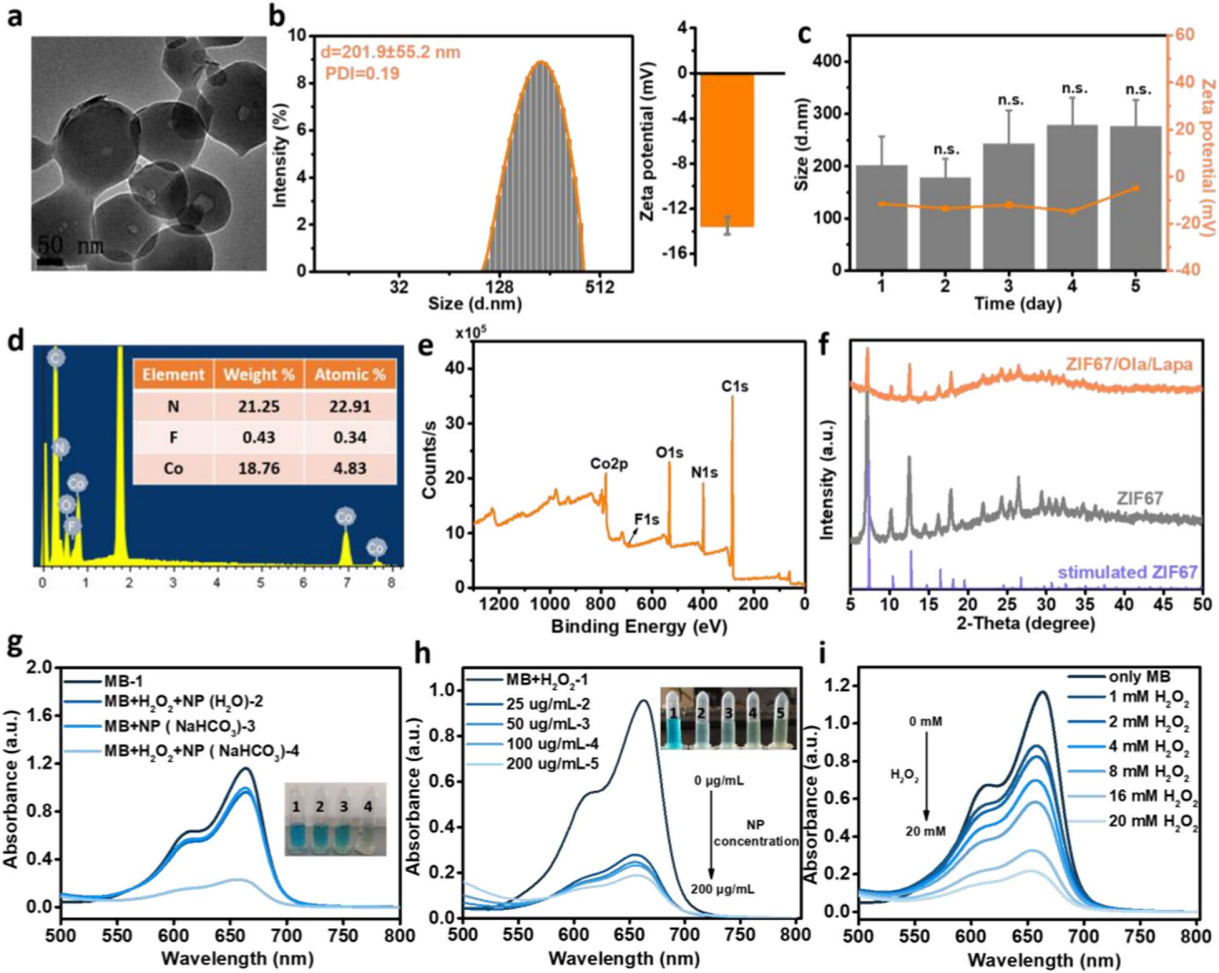
Characterizations of ZIF67/Ola/Lapa nanoparticles. **a** TEM image, **b** corresponding size distribution histogram (left) and zeta potential (right) of ZIF67/Ola/Lapa in water. **c** Hydrodynamic diameters and zeta potentials of ZIF67/Ola/Lapa dispersed in water (measured by DLS at indicated time points). n = 3, *n.s.* not significant. **d** EDS spectrum and corresponding element weight ratio (inset) of ZIF67/Ola/Lapa. **e** XPS spectrum of ZIF67/Ola/Lapa. **f** XRD patterns of ZIF67, and ZIF67/Ola/Lapa. **g** UV–vis absorption spectra and photograph (inset) of the solutions (water or 25 mM NaHCO_3_) containing MB, H_2_O_2_, and ZIF67/Ola/Lapa. **h** UV–vis absorption spectra of the solutions (25 mM NaHCO_3_) containing MB, 10 mM H_2_O_2_, and various concentrations of ZIF67/Ola/Lapa. **i** UV–vis adsorption spectra of the solutions (25 mM NaHCO_3_) containing MB, ZIF67/Ola/Lapa, and various concentrations H_2_O_2_

In this study, PARP inhibitor Ola and anticancer drug Lapa were encapsulated in ZIF67 via co-precipitation to prepare the ZIF67/Ola/Lapa nanoparticles. These nanoparticles were synthesized at room temperature by simply mixing aqueous solutions containing cobalt ions, 2-methylimidazole, Ola and Lapa. According to transmission electron microscopy (TEM) images, the obtained ZIF67/Ola/Lapa nanocomposites are monodispersed with defined spherical structure and mean diameter of 138.5 ± 15.9 nm (Fig. 1a and Additional file 1: Fig. S1), in line with the size of 132.2 ± 13.3 nm revealed by scanning electron microscope (SEM) images (Additional file 1: Fig. S2). As proved by dynamic light scattering (DLS), the hydrodynamic diameter of ZIF67/Ola/Lapa was calculated to be 201.9 ± 55.2 nm (polydispersity index = 0.19), which was little bigger than that shown in TEM images because of the existence of a hydrated layer in the aqueous environment [35]. Meanwhile, the strongly negative charge (− 12.0 ± 0.89 mV) of ZIF67/Ola/Lapa ensured that the nanocomposites were stable in biological environments (Fig. 1b). As expected, the ZIF67/Ola/Lapa nanoparticles behaved with excellent dispersibility and stability in water, saline and Dulbecco’s modified Eagle medium (DMEM) at least 5 days of incubation, demonstrated by negligible changes about the hydrodynamic size and zeta potential (Fig. 1c and Additional file 1: Fig. S3). The energy-dispersive X-ray (EDX) spectrum exhibited a clear distribution of Co, C, N, O, and F elements, which verified the successful loading of Ola because of the corresponding fluorine element (Fig. 1d), and the same conclusion could also be drawn from the spectrum of X-ray photoelectron spectroscopy (XPS) (Fig. 1e). Particularly, the Co 2p peaks were found to locate at 781 and 796.2 eV, assigning to Co 2p_3/2_ and Co 2p_1/2_, respectively (Additional file 1: Fig. S4a). In addition, the presence of an F 1s peak in the XPS spectrum was an indication of the successful encapsulation of Ola into the ZIF67/Ola/Lapa nanoparticles (Additional file 1: Fig. S4b).

X-ray diffraction (XRD) profiles of ZIF67 and ZIF67/Ola/Lapa retained well-defined diffraction peaks at 7.31° (0 1 1), 12.72° (1 1 2) and 16.45° (0 1 3) [36], which were identical to those of simulated ZIF67 (Fig. 1f). The UV–vis spectra of ZIF67/Ola/Lapa showed a characteristic absorption peak of Ola at 274 nm and obvious absorption at 444 nm attributed to Lapa, indicating the successful encapsulation of Ola and Lapa (Additional file 1: Fig. S5). The drug-loading capacity of ZIF67/Ola/Lapa was determined to be about 14.5 ± 0.45% for Ola and 31.1 ± 0.48% for Lapa based on the linear calibration curve between its concentration and the corresponding peak intensity, and UV–vis absorbance spectra of Ola, and Lapa before/after loading (Additional file 1: Figs. S6–S8 and Table S1). This one-pot drug-loading method achieved the encapsulation of Ola and Lapa in the internal part of ZIF67 simultaneously, facilitating multi-drug loading with high capacity and a simple procedure. Then, acid-responsive release profile of ZIF67/Ola/Lapa was investigated at pH 5.6 and pH 7.4 (Additional file 1: Fig. S9). Along with the reduction of pH value, the Ola and Lapa release percentage increased. After 24 h incubation, approximately 32% Lapa and 65% Ola was released from ZIF67/Ola/Lapa at pH 5.6, respectively.

### Detection of ·OH generation

The ·OH generating activity of ZIF67/Ola/Lapa-induced Fenton-like reaction was evaluated using the methylene blue (MB) assay, which can be oxidized by the highly reactive ·OH into colorlessness with the decline of maximum absorbance at about 665 nm. The results shown in Fig. 1g revealed that ZIF67/Ola/Lapa alone had a negligible effect on the absorbance of MB even when the incubation duration was extended to 2 h. In contrast, the color fading of MB solution was observed and the absorbance intensity of MB significantly reduced after treatment with ZIF67/Ola/Lapa and H_2_O_2_ in 25 mM NaHCO_3_ solution together, validating the superior ·OH production capability by the ZIF67/Ola/Lapa-driven Fenton chemistry. It was worthy to mention that the absorbance of MB had no detectable change with the same process in aqueous solution, which suggested the significance of HCO_3_^−^ in the ZIF67/Ola/Lapa-mediated Fenton-like reaction [36]. The UV–vis absorption spectra revealed that the variation in the concentration of nanoparticles had obvious effect on the decomposition of MB (Fig. 1h). Notably, the MB degradation enhanced with the increase of H_2_O_2_ concentration from 1 to 20 mM (Fig. 1i), and this H_2_O_2_ concentration-dependent Fenton-like effect provided the great possibility of enhanced ·OH generation by H_2_O_2_ self-supply from Lapa in vivo.

### Cellular uptake study

**Fig. 2:**
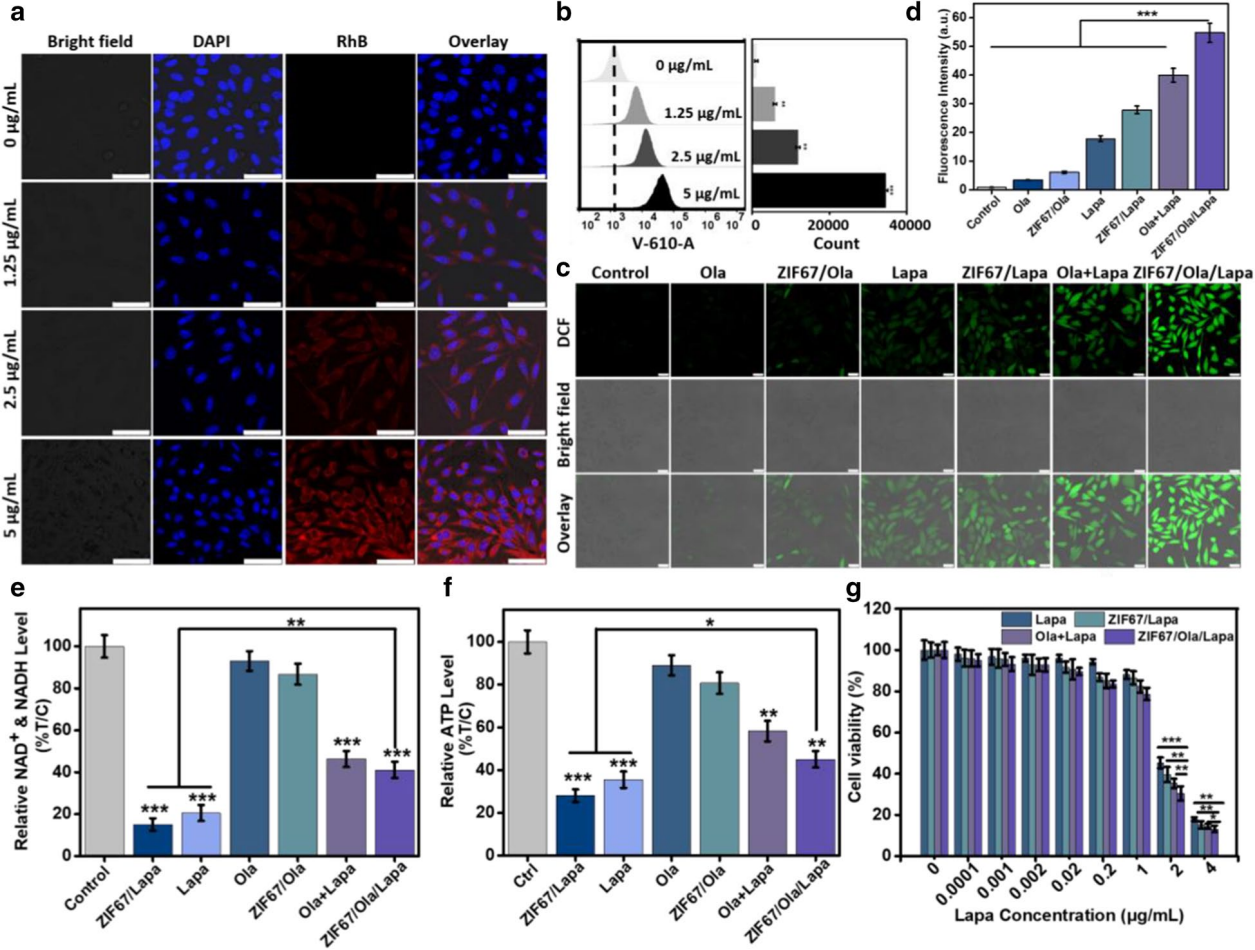
Cellular uptake and intracellular ROS production induced by ZIF67/Ola/Lapa nanoparticles. **a** CLSM images and **b** flow cytometry analysis of MDA-MB-231 cells incubated with various concentrations of ZIF67/Ola/Lapa for time periods within 4 h. Scale bar: 50 μm. **c** CLSM images and **d** corresponding quantitative fluorescence data of intracellular ROS generation treated with various drug formulations. Scale bar: 25 μm. (***p < 0.001). **e** Intracellular NAD^+^/NAD(P)H^+^ and **f** ATP level after 12 h incubation with different drugs. **g** In vitro cell cytotoxicity of different drug formulations against MDA-MB-231 cells after 24 h incubation. (*p < 0.05, **p < 0.01, ***p < 0.001)

Before evaluating the feasibility of ZIF67/Ola/Lapa for in vitro cancer cell killing, the cell uptake efficacy by MDA-MB-231 cells was evaluated firstly by confocal laser scanning microscopy (CLSM) and flow cytometric assay. From the microscope images (Fig. 2a), RhB labelled ZIF67/Ola/Lapa exhibited a concentration-dependent uptake and the red fluorescence enhanced with the increase of concentration. Consistently, results from flow cytometry also demonstrated the same gradual increasing trend of cellular uptake (Fig. 2b). In addition, the red fluorescence intensity inside the cells enhanced remarkably along with the increase of the incubation time (Additional file 1: Fig. S10), and quantitative flow cytometric analysis showed the internalization amount of ZIF67/Ola/Lapa at 4 h was up to 83.3%, indicating that ZIF67/Ola/Lapa could be internalized effectively by MDA-MB-231 cells (Additional file 1: Fig. S11).

### Intracellular amplified ROS production and in vitro toxicity

Based on the evaluation of cellular internalization, ZIF67/Ola/Lapa could be efficiently internalized by MDA-MB-231 cells. Thereafter, the probe 2′,7′-dichlorofluorescein diacetate (DCFH-DA) was employed to investigate the enhanced ·OH generation of ZIF67/Ola/Lapa in NQO1-overexpressing MDA-MB-231 cells [26]. As shown in Fig. 2c, cells exposed to ZIF67/Ola exhibited a weak green fluorescence, which could be ascribed to the low conversion efficiency of endogenous intracellular H_2_O_2_ by Co^2+^ to generate ·OH. In comparison, ZIF67/Lapa markedly increased ROS level, owing to the amplified oxidative stress mediated by Lapa. Expectedly, the treatment of ZIF67/Ola/Lapa resulted in the strongest green fluorescence intensity, which was about 8-, 2- and 1.3-fold higher than that of ZIF67/Ola, ZIF67/Lapa and Ola + Lapa (Fig. 2d), respectively, further confirming the self-augmented ·OH production. More importantly, there was no visible green fluorescence intensity in ZIF67/Ola/Lapa-treated HBL-100 normal cells because of the low NQO1 expression proved by PCR analysis (Additional file 1: Figs. S12, S13), suggesting that the ROS generation by ZIF67/Ola/Lapa nanosystem was dependent on NQO1 enzyme.

We further investigated the mechanism by which the synergy of Lapa and Ola caused the elevated H_2_O_2_ level. Dramatic NAD^+^/ATP losses occurred during Lapa-induced PARP hyperactivation, which was the main cause to restrain H_2_O_2_ formation [26]. So intracellular NAD^+^/NAD(P)H levels and ATP levels were then examined. Lapa and ZIF67/Lapa groups exhibited the significant reduction (around 80%) of total NAD^+^/NAD(P)H levels as a direct result of PARP hyperactivation. Addition of Ola to Lapa mitigated NAD^+^ losses obviously. Interesting, MDA-MB-231 cells treated with ZIF67/Ola/Lapa showed a higher level of NAD^+^/NAD(P)H (Fig. 2e). Similarly, ATP depletion mirrored changes in total NAD^+^ and NAD(P)H after Lapa alone and with Ola (Fig. 2f). These results indicated that Ola could prevent NAD^+^ and ATP loss, sustain NQO1 futile cycling of Lapa, and cause elevated H_2_O_2_ level.

**Fig. 3:**
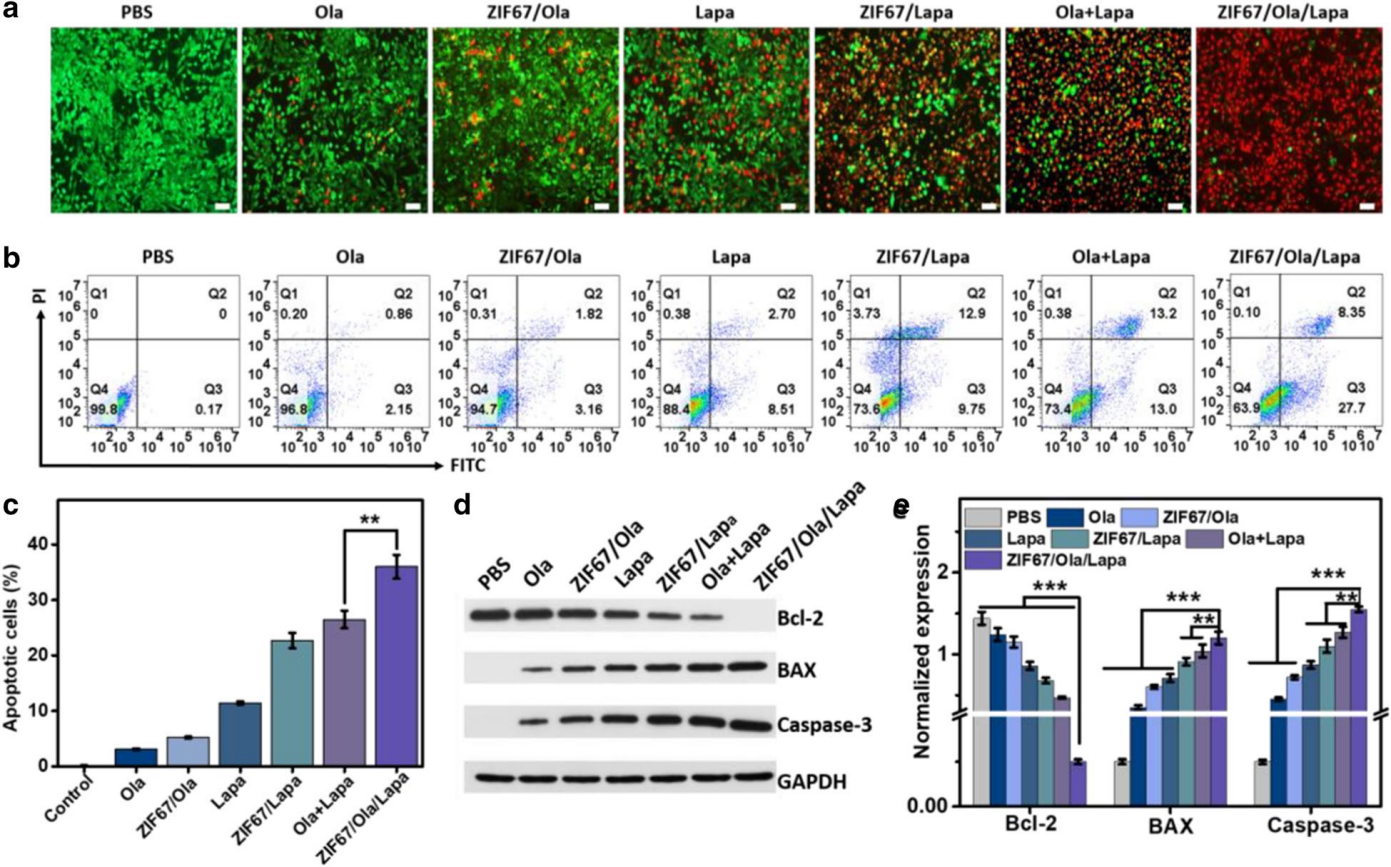
Cellular apoptosis induced by ZIF67/Ola/Lapa nanoparticles. **a** Fluorescence images of Calcein AM (green, live cells) and PI (red, dead cells) costained MDA-MB-231 cells after incubation with Ola, ZIF67/Ola, ZIF67/Lapa, Lapa, ZIF67/Lapa, Ola + Lapa and ZIF67/Ola/Lapa. Scale bar: 100 μm. **b** Cell apoptosis assay and **c** quantitative analysis of total apoptosis rate of MDA-MB-231 cells after incubating with different formulations for 6 h via Annexin V/PI staining. **d** Western blot results and **e** corresponding statistical data of the Bcl-2, BAX and Caspase-3 expressions in the MDA-MB-231 cells that received different treatments as indicated. (**p < 0.01, ***p < 0.001)

To quantitatively assess the in vitro chemodynamic efficiency of ZIF67/Ola/Lapa against MDA-MB-231 cells, cell counting kit-8 (CCK-8) assay was performed to test the cell viability. The cell viability exhibited negligible decrease when cells were treated with free ZIF67, even when the concentration reached up to 600 μg/mL (Additional file 1: Fig. S14), verifying that the conventional CDT efficacy was severely compromised by insufficient endogenous H_2_O_2_ in cancer cells. However, due to the autocatalytic production of H_2_O_2_ mediated by Lapa, appreciable cytotoxicity of Lapa and ZIF67/Lapa was observed (Fig. 2g). It was worth noting that ZIF67/Ola/Lapa exhibited a more serious toxicity on the MDA-MB-231 cells, which was attributed to the self-enhanced ·OH levels through the sustained H_2_O_2_ generation synergized by Ola, Lapa and Fenton-like reaction. Additionally, it was found that ZIF67/Ola/Lapa nanoparticles could result in the significant (p < 0.01) cytotoxicity towards NQO1-overexpressing A549 lung cancer cells compared to NQO1-deficient A549 cancer cells pretreated with Dicoumarol (Additional file 1: Fig. S15). Intriguingly, ZIF67/Ola/Lapa also did not show appreciable cytotoxicity to non-cancerous HBL-100 cells (Additional file 1: Fig. S16), further confirming that the ability of ZIF67/Ola/Lapa to specifically kill tumor cells overexpressing-NQO1. To observe the distribution of live and dead cells more intuitively, MDA-MB-231 cells were treated with different samples and then costained with calcein acetoxymethyl ester (Calcein-AM) and propidium iodide (PI). The live and dead cells were stained with green and red fluorescence, respectively. As shown in Fig. 3a, the cells without any treatment were growing very well, while a weak damage was observed for the cells treated with Ola, ZIF67/Ola and Lapa. Meanwhile, a majority of dead cells were observed when treated with ZIF67/Lapa and Ola + Lapa. Significantly, almost all the cells are dead after treated with ZIF67/Ola/Lapa, which was considered to be induced by the cooperative chemodynamic therapies. The apoptotic and necrotic cells were further analyzed examined using the annexin V-FITC/PI apoptosis detection kit by flow cytometry. Notably, the ZIF67/Ola/Lapa-treated MDA-MB-231 cells had the highest apoptosis ratio of 36.05%, 5.93-, 1.59- and 1.38-fold higher than that of ZIF67/Ola, ZIF67/Lapa, and Ola + Lapa groups (Fig. 3b, c). To further illuminate the possible mechanisms of cell apoptosis pathway, the expression of Bcl-2 (anti-apoptotic protein), Bax and Caspase-3 (pro-apoptotic protein) in tumor cells was evaluated by western blotting (Fig. 3d, e). After Lapa treatment, the expressions of Bax and Caspase-3 were moderately up-regulated, suggesting that H_2_O_2_-induced apoptosis was activated through NQO1-catalyzed ineffective cycle of Lapa. Besides, Ola + Lapa treatment led to a more remarkable increase in the expression of these apoptosis-related proteins, suggesting the capability of Ola to enhance Lapa-mediated apoptosis by inhibiting DNA repair. Undoubtedly, the advantage of combination therapy was amplified by ZIF67/Ola/Lapa, which resulted in the most significant upregulation of Bax and Caspase-3 as well as the most obvious reduction in Bcl-2 expression. These results confirmed that the ZIF67/Ola/Lapa nanosystem, serving as an enhanced chemodynamic nanoagent with self-supplied H_2_O_2_, possessed potent anticancer activity.

### In vivo biodistribution study

**Fig. 4:**
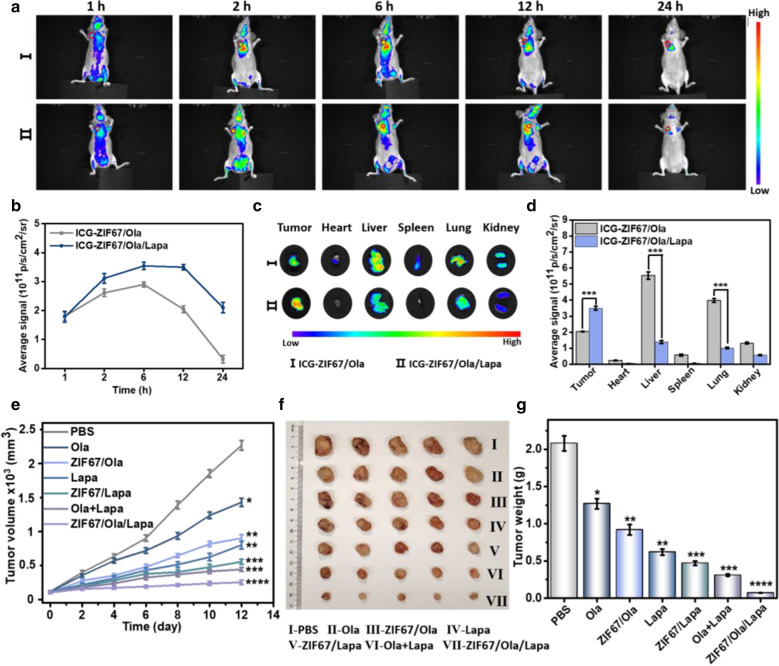
In vivo biodistributions and enhanced CDT of ZIF67/Ola/Lapa nanoparticles against tumor. **a** In vivo distributions of ICG-ZIF67/Ola and ICG-ZIF67/Ola/Lapa after intravenous administration. The red circle represents the tumor. **b** Fluorescence intensity of the tumor tissues at various time points. **c** Representative fluorescence images of dissected organs and tumors at 12 h. **d** Semiquantitative analysis of fluorescence intensity of the major organs and tumor tissues collected at 12 h. (n = 3, ***p < 0.001). **e** Tumor growth curves of mice after various treatments. **f** Digital photos of excised tumor from the mice and **g** the average tumor weight after 12 days of treatment. (n = 5, *p < 0.05, **p < 0.01, ***p < 0.001)

For validating the tumor-targeting of the nanosystem, in vivo distribution of ZIF67/Ola/Lapa in MDA-MB-231 tumor-bearing mice was systematically studied. For the mice treated with ICG-labelled ZIF67/Ola, the weak fluorescence of tumor site disappeared rapidly when the time extended to 24 h. By contrast, the fluorescence signal of ICG-ZIF67/Ola/Lapa occurred in tumor site at 1 h post-injection, gradually increased and reached the maximum at 6 h, even retained notably higher fluorescence intensity in tumor tissues within 24 h post-injection, suggesting a long retention ability at tumor site (Fig. 4a, b). At 12 h post-injection, the tumors and main organs in each group were excised for further ex vivo fluorescence imaging (Fig. 4c). It should be noted that the fluorescence intensity of tumor treated with ICG-ZIF67/Ola/Lapa was almost 1.8 times higher than that of ICG-ZIF67/Ola after 12 h of administration (Fig. 4d), further demonstrating the good tumor accumulation capacity. The ex vivo fluorescence images also revealed that ICG-ZIF67/Ola/Lapa was metabolized mainly by the liver and kidneys. These results indicated that, the prepared ZIF67/Ola/Lapa could preferentially and efficiently accumulate in tumor tissues with prolonged blood circulation, which could be attributed to the overexpressed NQO1 enzyme in tumor tissues.

### In vivo antitumor efficiency

The excellent chemodynamic efficacy and efficient tumor accumulation of ZIF67/Ola/Lapa motivated us to conduct the in vivo therapeutic evaluation via MDA-MB-231 tumor-bearing mice. According to the tumor growth curves in Fig. 4e, both Ola and ZIF67/Ola treatments failed to effectively suppress tumor growth, while Lapa and ZIF67/Lapa had obvious inhibitory effects on tumor growth, attributable mainly to the generation of H_2_O_2_ mediated by Lapa. As expected, the most effective inhibition was achieved in mice treated with ZIF67/Ola/Lapa compared to all of the groups. In this group, the inhibition rate of the tumor reached 88.7% after 12 days of treatment, while 75.3% and 80.3% of inhibition rates were obtained for the groups treated with ZIF67/Lapa and Ola + Lapa, respectively, demonstrating the superior tumor growth suppression effect of ZIF67/Ola/Lapa. And the weight of excised solid tumors on day 12 also exhibited a trend identical to that of tumor sizes (Fig. 4f, g). This superior tumor inhibition effect of ZIF67/Ola/Lapa could be ascribed to the extensive intratumoral ·OH levels by the cooperative therapeutic efficacy including the sustained H_2_O_2_ generation synergized by Ola and Lapa, as well as the amplified CDT induced by ZIF67.

**Fig. 5:**
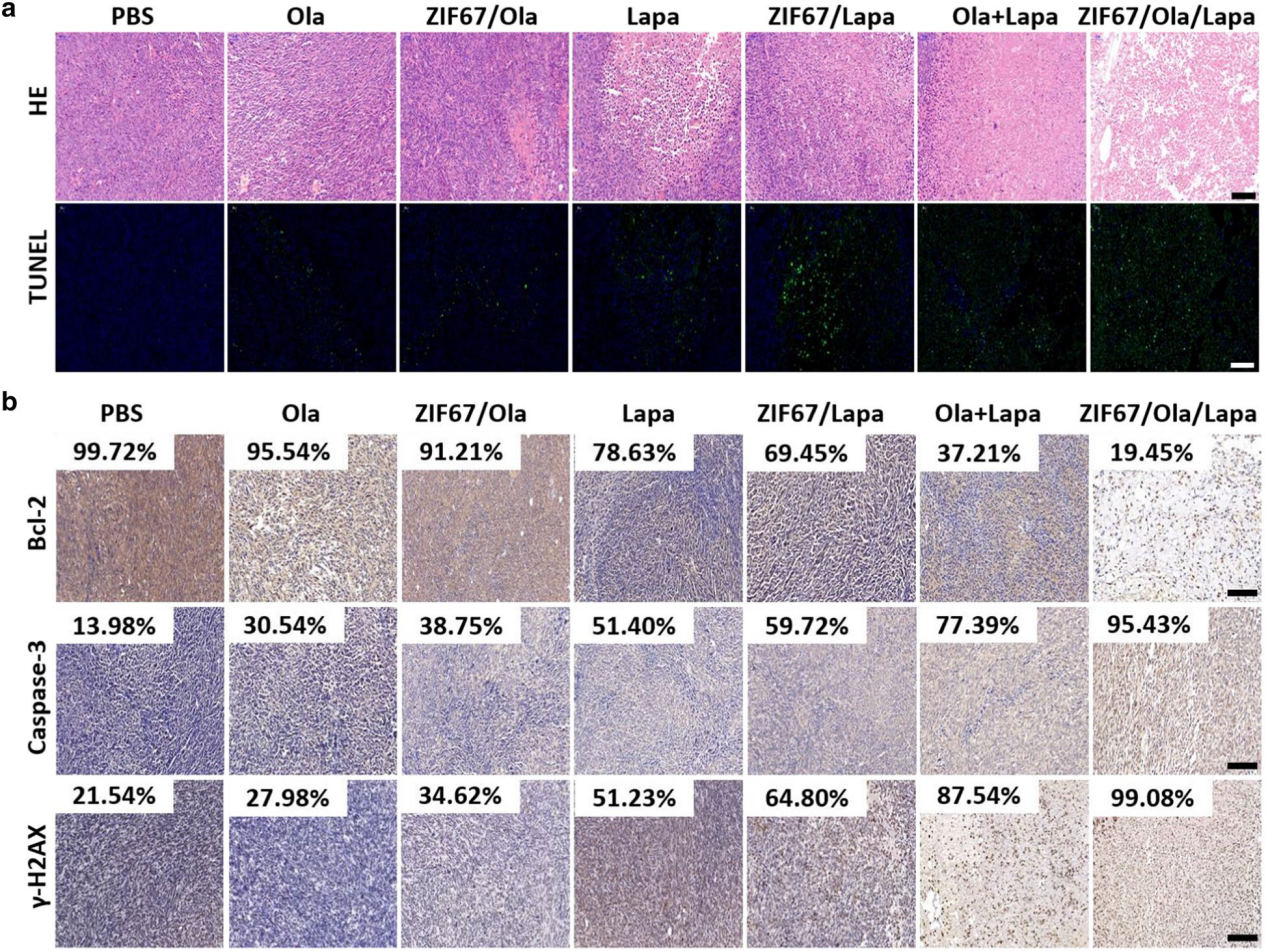
The anti-tumor effect induced by ZIF67/Ola/Lapa nanoparticles. **a** H&E staining images and TUNEL staining images for tumor slices collected after different treatments. Scale bar: 100 μm. **b** Immunohistochemically stained images of tumors of Bcl-2, Caspase-3, and γ-H2AX. Scale bar: 100 μm

The high anticancer activity of ZIF67/Ola/Lapa was also confirmed by the histological analysis of tumor tissues with hematoxylineosin (H&E) staining. Tumor tissue in the ZIF67/Ola/Lapa group suffered the most severe damage, as revealed by the distinct cell shrinkage and chromatin condensation in H&E observation (Fig. 5a). Furthermore, TUNEL images also showed a notable increase in the proportion of TUNEL-positive cells induced by ZIF67/Ola/Lapa. As exhibited by immunohistochemical (IHC) staining, the expressions of apoptosis related proteins γ-H2AX and Caspase-3 were significantly increased after ZIF67/Ola/Lapa treatment, while the antiapoptotic protein Bcl-2 was significantly inhibited (Fig. 5b). Such results were in accordance with the therapeutic outcome of in vivo antitumor experiment.

### In vivo antitumor mechanism

**Fig. 6:**
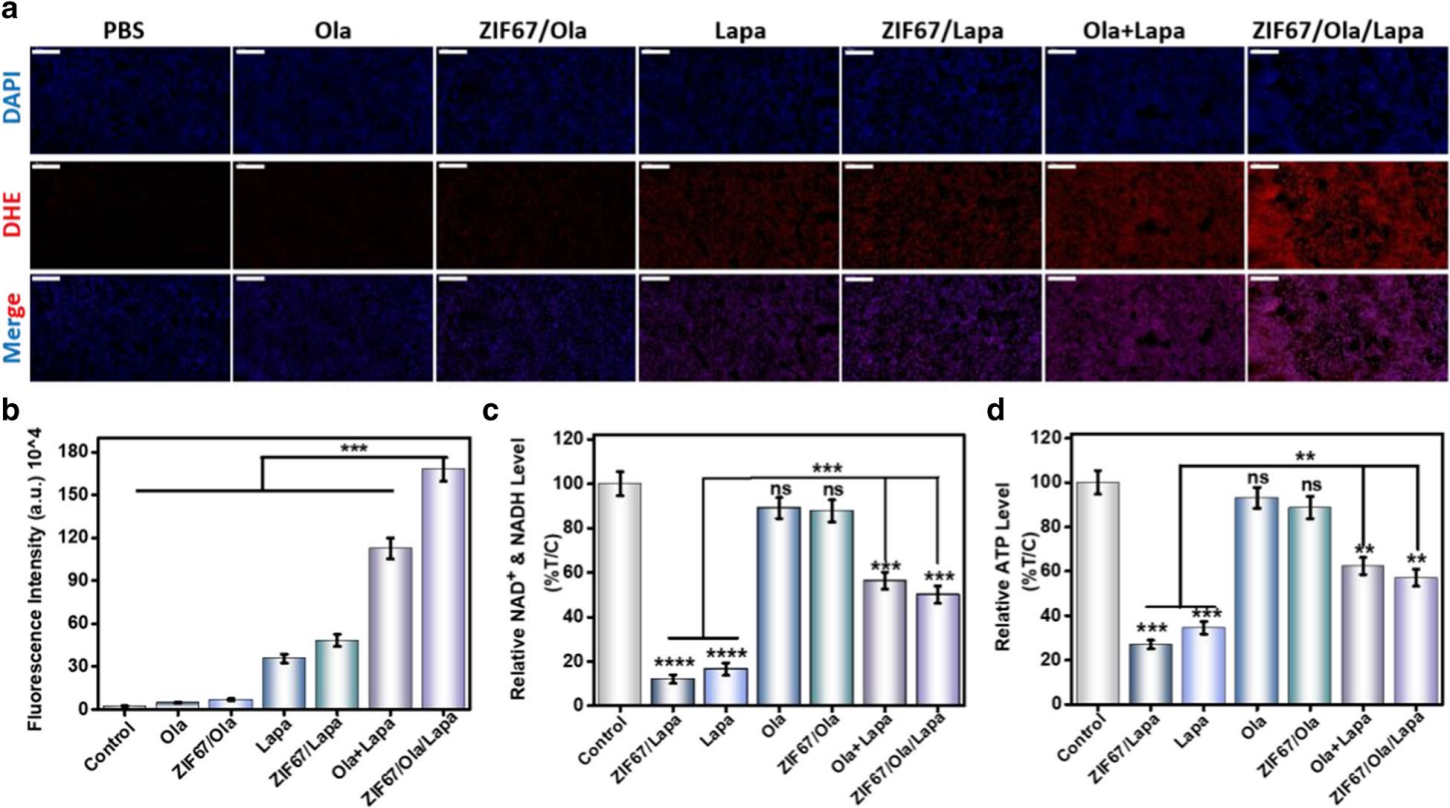
The anti-tumor mechanism induced by ZIF67/Ola/Lapa nanoparticles. **a** Immunofluorescence staining of DHE to detect intratumoral hydroxyl radicals after different treatments. Nuclei (blue) were stained with DAPI. ROS (red) were stained with DHE. Scale bar: 100 μm. **b** Semiquantitative analysis of fluorescence signals with DHE immunofluorescence staining. **c** NAD^+^/NAD(P)H^+^ and **d** ATP level from tumor tissues with different treatments. (****p < 0.0001, ***p < 0.001)

To gain further insight into the mechanism of excellent anticancer performance of ZIF67/Ola/Lapa, the tumors were collected for ROS staining via an ex vivo DHE staining assay. Weak red fluorescence could be detected in the control group or tumor treated with ZIF67/Ola, and relatively brighter fluorescence appeared in Lapa- and ZIF67/Lapa-treated group, which was owing to the on-demand generation of a large amount of H_2_O_2_ by Lapa. Importantly, the ZIF67/Ola/Lapa treated group markedly strengthened the red fluorescence intensity (Fig. 6a, b), and this strongest ROS generation in H_2_O_2_ self-supplying CDT gave reason to the excellent antitumor effect of ZIF67/Ola/Lapa. The elevated ROS levels were related to the continuous NQO1 futile redox cycling synergized by Lapa and Ola. For further clarification, NAD^+^/NAD(P)H levels and ATP levels from tumor tissues were accordingly examined. In contrast to the significant loss of NAD^+^/NAD(P)H and ATP in the Lapa and ZIF67/Lapa groups due to Lapa-induced PARP hyperactivation, the ZIF67/Ola/Lapa group remarkably increased the NAD^+^/NAD(P)H and ATP levels to 56.3%, and 57.8% (Fig. 6c, d), respectively, indicating that the addition of Ola could mitigate NAD(P)H consumption along with ATP depletion, and enhance ROS formation because of the sustained NQO1 futile redox cycling.

### Biosafety evaluation in vivo

The investigation of systemic toxicity of ZIF67/Ola/Lapa was crucial for possible biomedical application. Importantly, no remarkable weight loss changes were observed during a 12-day observation period (Additional file 1: Fig. S17), proving that ZIF67/Ola/Lapa had low adverse effects in vivo. Additionally, H&E staining images of the major organs showed no detectable damage or inflammatory lesions after treatment with ZIF67/Ola/Lapa (Additional file 1: Fig. S18). Furthermore, no significant changes occurred about the standard biochemical indexes including alanine aminotransferase (ALT) and creatinine (CREA) levels in the ZIF67/Ola/Lapa-treated mice compared to the control group (Additional file 1: Fig. S19), suggesting the healthy liver and kidney functions. Moreover, white blood cells (WBC) and hemoglobin (HGB) were found to be at normal levels. These results pointed out that the synthesized ZIF67/Ola/Lapa nanoparticles with advanced tumor inhibition effect and good biocompatibility are promising candidates for cooperative cancer therapy.

## Conclusions

In summary, the PARP inhibitor Ola and NQO1-bioactivatable drug Lapa were assembled with cobalt-based metal–organic framework (ZIF67) to construct the nanocatalytic system (ZIF67/Ola/Lapa) for solving the insufficient endogenous concentration of H_2_O_2_ in cancer cells and the negative effect of PARP hyperactivation on the CDT efficacy. Ola and Lapa molecules delivered intracellularly by ZIF67/Ola/Lapa could synergistically achieve the tumor-specific sustainable ·OH production through NQO1-dependent futile redox cycling. In turn, the produced H_2_O_2_ was effectively catalyzed by ZIF67, via Fenton-like reaction, to induce highly oxidative ·OH for amplified CDT. Both in vitro and in vivo studies demonstrated that ZIF67/Ola/Lapa presented remarkable capability in the inhibition of NQO1 overexpressed MDA-MB-231 cells and tumor progression without toxicity to normal tissues owing to the low NQO1 expression. This study provides a new paradigm for the design of multifunctional CDT agents taking advantage of the combination of PARP inhibitor and NQO1-bioactivatable drug.

## Supplementary Information

**Additional file 1. Fig. S1.** TEM corresponding size distribution histogram of ZIF67/Ola/Lapa. **Fig. S2.** SEM image and  corresponding size distribution histogram of ZIF67/Ola/Lapa nanoparticles. **Fig. S3.** Hydrodynamic diameters of ZIF67/Ola/Lapa dispersed in  saline and  DMEM. **Fig. S4**. XPS high-resolution spectrum of  Co 2p and  F1s. **Fig. S5.** UV–vis absorption spectra of the solutions containing ZIF67, ZIF67/Ola, ZIF67/Lapa and ZIF67/Ola/Lapa. **Fig. S6.**  UV–vis absorption spectra of the solutions containing different concentrations of Ola and  its standard curve. **Fig. S7.** UV–vis absorption spectra of the solutions containing different concentrations of Lapa and its standard curve. **Fig. S8.** UV-vis absorbance spectra of Ola, and Lapa before and after loading. **Fig. S9.** Drug release behavior of Lapa and  Ola at different pH. **Fig. S10.** CLSM images of MDA-MB-231 cells incubated with ZIF67/Ola/Lapa for different time periods. **Fig. S11.** Flow cytometry of MDA-MB-231 cells incubated with ZIF67/Ola/Lapa for different time periods. **Fig. S12.** CLSM images of intracellular ROS generation treated with ZIF67/Ola/Lapa in HBL-100 normal cells. **Fig. S13.** PCR analysis of NQO1 expression level of HBL-100 normal cells and MDA-MB-231 breast cancer cells. **Fig. S14.** The cytotoxicity of  ZIF67, Ola and ZIF67/Ola towards MDA-MB-231 cells. **Fig. S15.** The cytotoxicity of ZIF67/Ola/Lapa towards A549 cells or A549 cells pretreated with Dicoumarol. **Fig. S16.** The cytotoxicity of ZIF67/Ola/Lapa towards HBL-100 normal cells. **Fig. S17.** Body weight of MDA-MB-231 tumor-bearing mice under different treatments. **Fig. S18.** H&E stained images of major organs obtained from different groups. **Fig. S19.** Blood biochemistry data including numbers of ALT, CREA, WBC, and HGB. **Table S1:** The detailed data of drug loading capacity.

## Data Availability

The data are available in the main manuscript, additional information files, and from the corresponding authors upon reasonable request.
