# Supplementary material for: A self-amplified nanocatalytic system for achieving “1 + 1 + 1 > 3” chemodynamic therapy on triple negative breast cancer
Source: J Nanobiotechnology. 2021 Sep 4;19:261. doi: 10.1186/s12951-021-00998-y (PMC8418045; doi:10.1186/s12951-021-00998-y)
Supplement: Supplementary file 1 — Additional file 1. Fig. S1. TEM corresponding size distribution histogram of ZIF67/Ola/Lapa. Fig. S2. SEM image and corresponding size distribution histogram of ZIF67/Ola/Lapa nanoparticles. Fig. S3. Hydrodynamic diameters of ZIF67/Ola/Lapa dispersed in saline and DMEM. Fig. S4. XPS high-resolution spectrum of Co 2p and F1s. Fig. S5. UV–vis absorption spectra of the solutions containing ZIF67, ZIF67/Ola, ZIF67/Lapa and ZIF67/Ola/Lapa. Fig. S6. UV–vis absorption spectra of the solutions containing different concentrations of Ola and its standard curve. Fig. S7. UV–vis absorption spectra of the solutions containing different concentrations of Lapa and its standard curve. Fig. S8. UV-vis absorbance spectra of Ola, and Lapa before and after loading. Fig. S9. Drug release behavior of Lapa and Ola at different pH. Fig. S10. CLSM images of MDA-MB-231 cells incubated with ZIF67/Ola/Lapa for different time periods. Fig. S11. Flow cytometry of MDA-MB-231 cells incubated with ZIF67/Ola/Lapa for different time periods. Fig. S12. CLSM images of intracellular ROS generation treated with ZIF67/Ola/Lapa in HBL-100 normal cells. Fig. S13. PCR analysis of NQO1 expression level of HBL-100 normal cells and MDA-MB-231 breast cancer cells. Fig. S14. The cytotoxicity of ZIF67, Ola and ZIF67/Ola towards MDA-MB-231 cells. Fig. S15. The cytotoxicity of ZIF67/Ola/Lapa towards A549 cells or A549 cells pretreated with Dicoumarol. Fig. S16. The cytotoxicity of ZIF67/Ola/Lapa towards HBL-100 normal cells. Fig. S17. Body weight of MDA-MB-231 tumor-bearing mice under different treatments. Fig. S18. H&E stained images of major organs obtained from different groups. Fig. S19. Blood biochemistry data including numbers of ALT, CREA, WBC, and HGB. Table S1: The detailed data of drug loading capacity. [file 12951_2021_998_MOESM1_ESM.docx]

**Additional file 1**

**A Self-Amplified Nanocatalytic System for Achieving "1+1+1>3" Chemodynamic Therapy on Triple Negative Breast Cancer**

Lulu Zhou^a,1^, Jinjin Chen^a,b,1^, Yunhao Sun^c^, Keke Chai^a^, Zhounan Zhu^a^, Chunhui Wang^a^,  Mengyao Chen^a^, Wenmei Han^a^, Xiaochun Hu^a^, Ruihao Li^a^,  Tianming Yao^a^, Hui Li^a^, Chunyan Dong^a,^* and Shuo Shi^a,^*

^a^ Shanghai Key Laboratory of Chemical Assessment and Sustainability, School of Chemical Science and Engineering, Breast Cancer Center, Shanghai East Hospital, Tongji University, Shanghai, 200092, P. R. China.

^b^ Department of Oncology, The Fourth Affiliated Hospital of Nantong University, First People's Hospital of Yancheng, Yancheng, Jiangsu, P. R. China.

^c^ Department of Thoracic surgery, First People's Hospital of Yancheng, affiliated to medical college of Nanjing university, Yancheng, Jiangsu, P. R. China.

*Corresponding authors.

E-mail: shishuo@tongji.edu.cn (S. Shi); cy_dong@tongji.edu.cn (C. Dong).

^1^ L. Zhou and J. Chen contributed equally to this work.

**Materials and methods**

**Materials**

Co(NO_3_)_2_•6H_2_O, methylene blue (MB), Rhodamine B (RhB), ICG and H_2_O_2_ were acquired from Aladdin Corporation (Shanghai, China). 2-methylimidazole (MeIm) was acquired from MERYER CO., LTD. Olaparib (Ola) and β-Lapachone (Lapa) was purchased from MedChemExpress (Monmouth Junction, NJ, USA). High glucose Dulbecco’s modified Eagle’s medium (DMEM), 1% penicillin–streptomycin (PS), 0.25% trypsin–EDTA, phosphate and phosphate buffered saline (PBS) were obtained from Jiangsu KeyGEN BioTech Corp.,Ltd. Fetal bovine serum (FBS) was purchased from Gibco Invitrogen.

**Characterizations**

The crystal phase of the samples was investigated by X-ray powder diffraction (XRD; Bruker D8 Advance X-ray diffractometer) with Cu Kα radiation (λ = 0.154 nm). SEM image, elemental mapping and energy dispersive spectroscopy were conducted on Hitachi-4800. TEM images were performed on JEM-2100 (JEOL, Japan) at an accelerating voltage of 200 KV. UV-visible absorption spectra were recorded by a Hitachi U-2900 Spectrophotometer. DLS and ζ-potential of the samples were measured by Litesizer TM 500 (Anton Paar). X-ray photoelectron spectra (XPS) were recorded on a Thermo ESCALAB 250Xi using Al Kα (1486.6 eV) as the excitation source. Confocal laser scanning microscopy (CLSM) images were recorded on a confocal microscope (Zeiss Axio-Imager LSM-880). The flow cytometry data was obtained by BD FACS Aria II.

**Drug loading**

The one-pot drug loading method was applied to encapsulate Ola and Lapa together during the preparation of ZIF67. The residual of Ola and Lapa in the supernatant was measured using the Hitachi U-2900 Spectrophotometer by the absorbance at 274 nm and 444 nm, respectively. The drug loading capacity was calculated through the following equation [1]:

Drug loading capacity = *W*_Fed drug_-*W*_drug in supernatant_/*W*_ZIF67/Ola/Lapa NPs_ x 100%

where *W*_Fed drug_ is the initial amount of fed drug, *W*_drug in supernatant_ is the amount of drug in all the supernatant collected after centrifugations, and *W*_ZIF67/Ola/Lapa NPs_ is the amount of as-synthesized ZIF67/Ola/Lapa NPs.

**Cell culture**

MDA-MB-231 cells were cultured in high glucose DMEM (FB25015, Clarkbio, Shanghai, China) containing 10% fetal bovine serum and 1% penicillin-streptomycin solution under a humidified 5% carbon dioxide at 37°C.

**Cellular uptake behaviors of ZIF67/Ola/Lapa nanoparticles**

The cellular uptake experiment was carried out using CLSM and flow cytometry, according to different concentrations and time points. For dose-dependent uptake experiments, MDA-MB-231 cells were seeded in 6-well plates at a density of 5×10^5^ cells per well and cultured for 24 h, ZIF67/Ola/Lapa with different concentrations (1.25, 2.5 and 5 µg/mL) were added into dishes and incubated with cells for 4 h at 37°C. Similarly, in time-dependent cellular uptake analysis, MDA-MB-231 cells were incubated in 6-well plates with a density of 5×10^5^ cells per well for 24 h. Then cells were washed with PBS and replaced with ZIF67/Ola/Lapa NPs (5 μg/mL) at different time points (1, 2, 4 and 6 h) at 37°C. Then The cells were harvested and dispersed in PBS for uptake study by flow cytometry.

**Determination of ROS generation in vitro**

The intracellular ROS generation of ZIF67/Ola/Lapa NPs was detected using Reactive Oxygen Species Assay Kit based on DCFH-DA (2,7-dichlorodihydrofluorescein diacetate). MDA-MB-231 cells were plated on confocal culture dishes. After 24 h, cells were treated with Ola, Lapa, Ola+Lapa, ZIF67/Ola, ZIF67/Lapa and ZIF67/Ola/Lapa (equivalent to 1 µg/mL of Lapa) for 4 h at 37°C. The cells which were incubated with PBS in dark were as the control groups. After 4 h, the medium was removed and DCFH was added (final concentration 1×10^-6^ M) and incubated for 30 min. Finally, all the cells were viewed by CLSM with laser at 488 nm.

**Live/dead cell staining assay**

MDA-MB-231 cells with a density of 10^5^ cells per well were cultured in a 6-well plate for 24 h to allow the attachment of cells. After cells were washed twice by PBS solution, Ola, Lapa, Ola+Lapa, ZIF67/Ola, ZIF67/Lapa and ZIF67/Ola/Lapa (equivalent to 2 µg/mL of Lapa) were added to above culture medium. Altering incubating for 24 h, the cells were stained with Calcein-AM (4 × 10^−6^ M) and PI solutions (4 × 10^−6^ M) in PBS buffer solution and incubated for 30 min. Finally, the cells were washed several times with PBS buffer solution and observed by Inverted fluorescence microscope (Nikon ECLIPSE Ti) to examine their live/dead status. The Calcein-AM and PI were excited with lasers at 488 and 543 nm, respectively.

**Cell apoptosis measurement**

The cell apoptosis analysis against MDA-MB-231 cells was performed by utilizing a flow cytometric assay of Annexin V-FITC and PI containing. MDA-MB-231 cells were seeded in 6-well plates and were treated with Ola, Lapa, Ola+Lapa, ZIF67/Ola, ZIF67/Lapa and ZIF67/Ola/Lapa (equivalent to 2 µg/mL of Lapa). After co-incubation for 6 h, the medium was replaced with fresh medium. MDA-MB-231 cells were stained with Annexin V-FITC/PI for 20 min. Subsequently, the cells were collected for flow cytometry measurement.

**Animal models**

Female Balb/c mice (4-5 weeks) were purchased from Shanghai Laboratory Animal Center (SLAC, Shanghai, China) and bred in a sterilized, specific pathogen-free (SPF) Lab of Tongji University. Then the MDA-MB-231 cells were injected into the right mammary fat pad of five-week-old female BALB/c mice. All animal procedures conformed to the Guide for the Care and Use of Laboratory Animals.

**References**

1. Cai X, Jia X, Gao W, Zhang K, Ma M, Wang S, et al. A Versatile Nanotheranostic Agent for Efficient Dual-Mode Imaging Guided Synergistic Chemo-Thermal Tumor Therapy. Adv. Funct. Mater., 2015, 25, 2520-9.


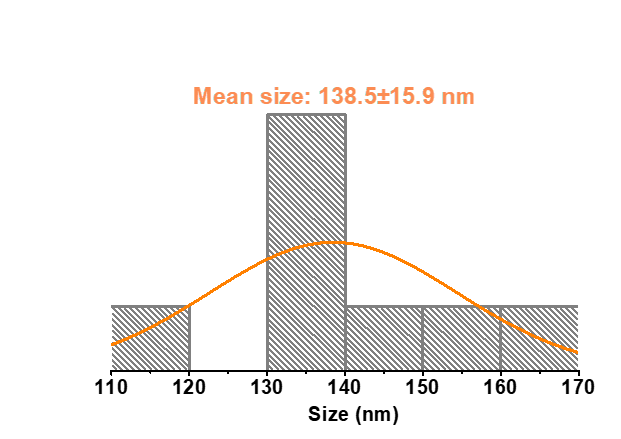


Fig. S1. TEM corresponding size distribution histogram of ZIF67/Ola/Lapa.


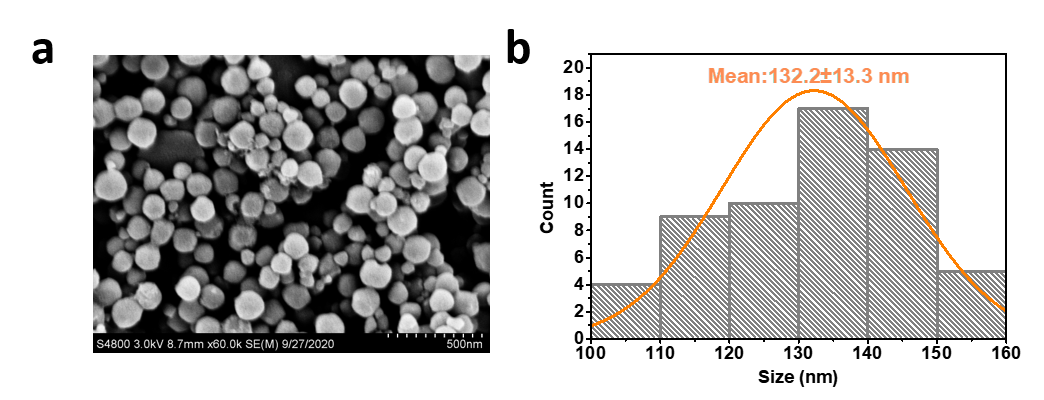


Fig. S2. (a) SEM image and (b) corresponding size distribution histogram of ZIF67/Ola/Lapa nanoparticles.


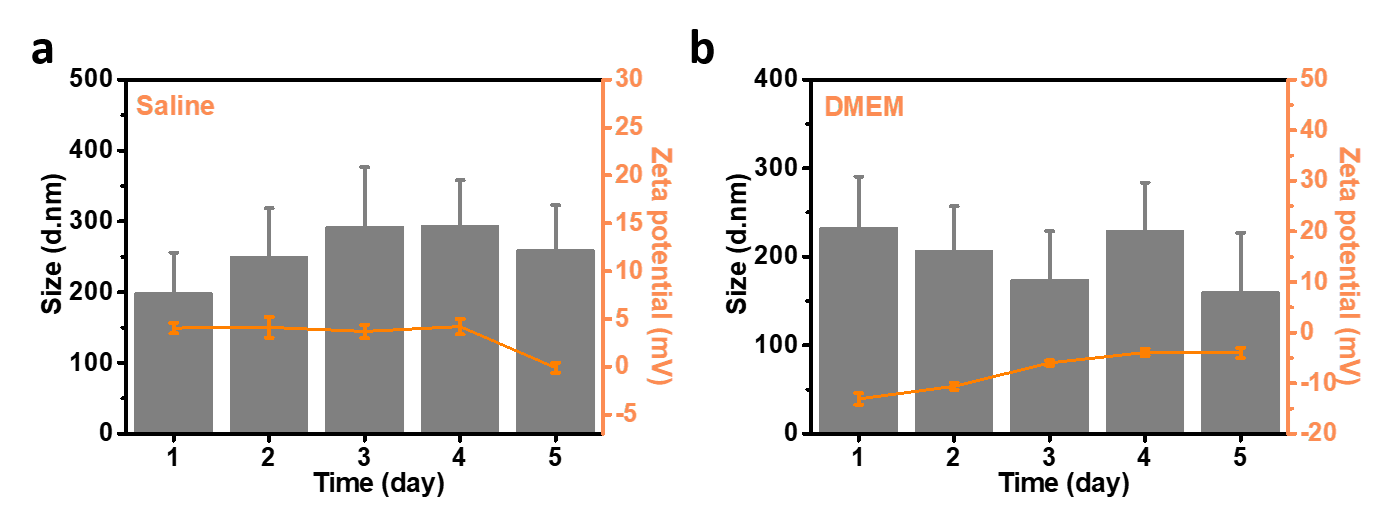


Fig. S3. Hydrodynamic diameters of ZIF67/Ola/Lapa dispersed in (a) saline and (b) DMEM (measured by DLS at indicated time points).


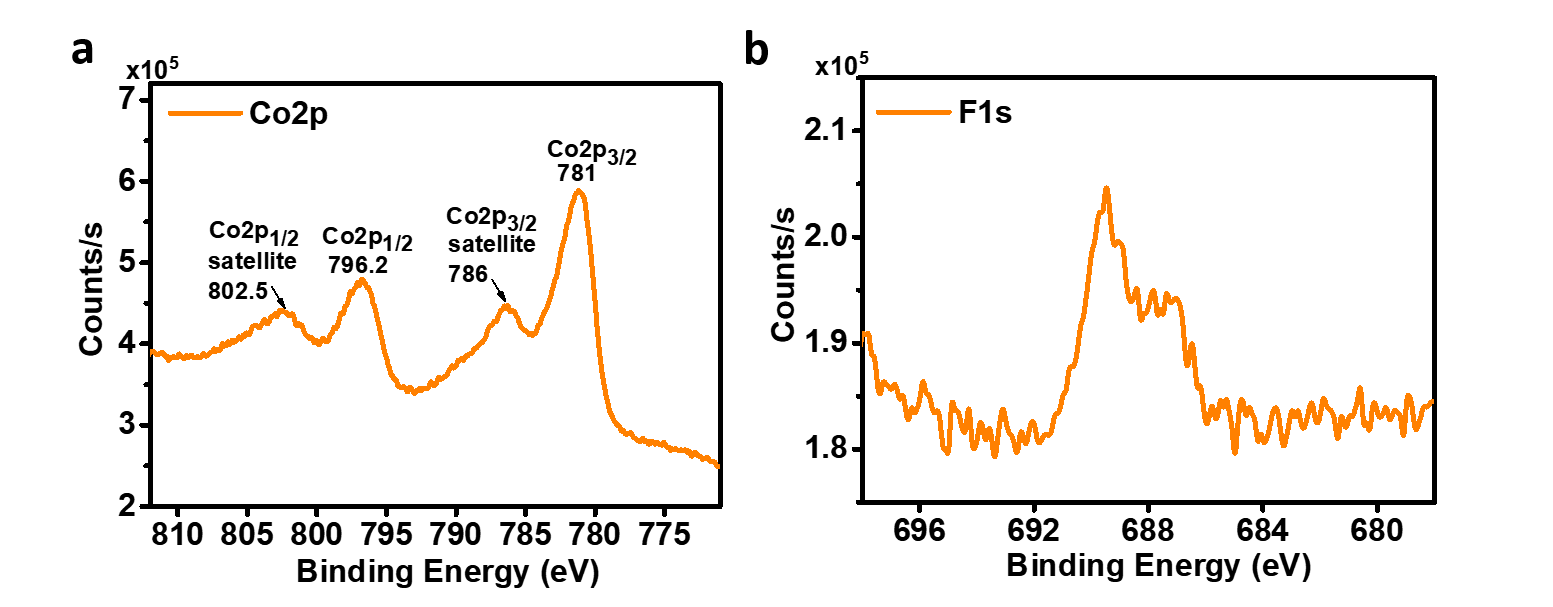


Fig. S4. XPS high-resolution spectrum of (a) Co 2p and (b) F1s.


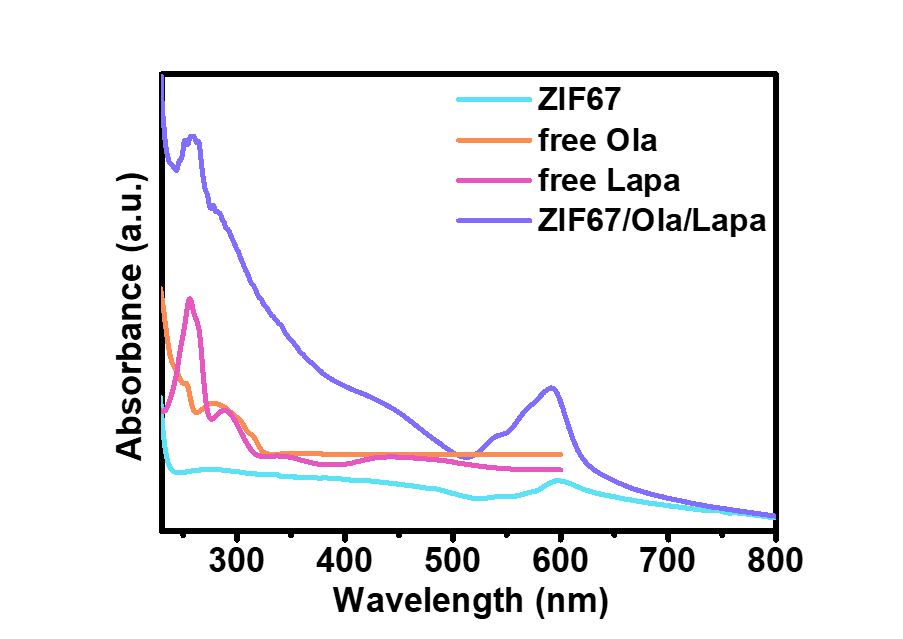


Fig. S5. UV–vis absorption spectra of the solutions containing ZIF67, ZIF67/Ola, ZIF67/Lapa and ZIF67/Ola/Lapa.


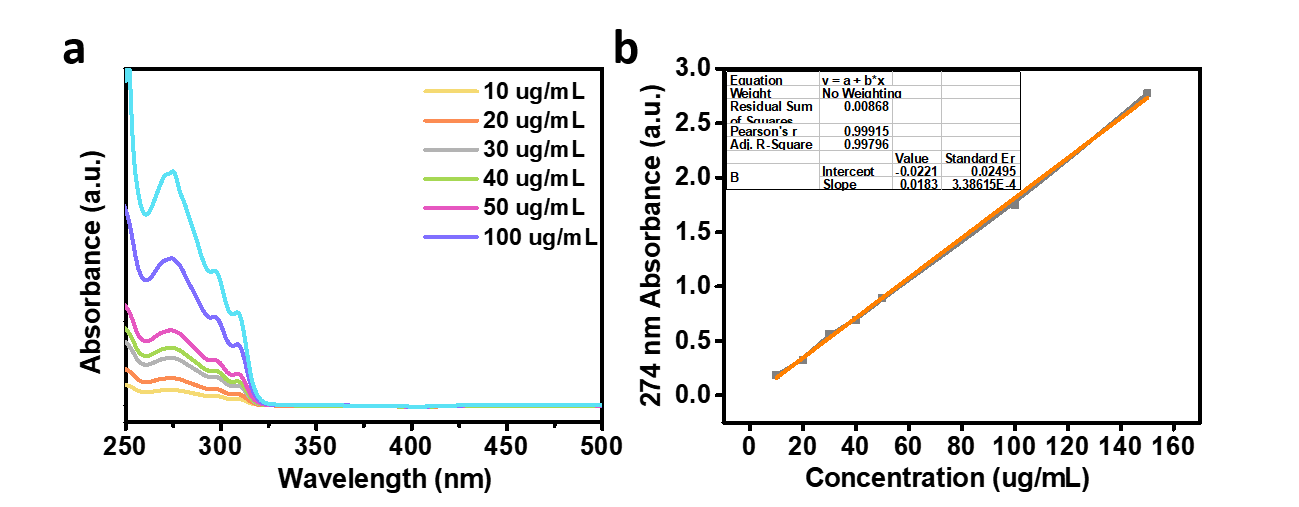


Fig. S6. (a) UV–vis absorption spectra of the solutions containing different concentrations of Ola and (b) its standard curve.


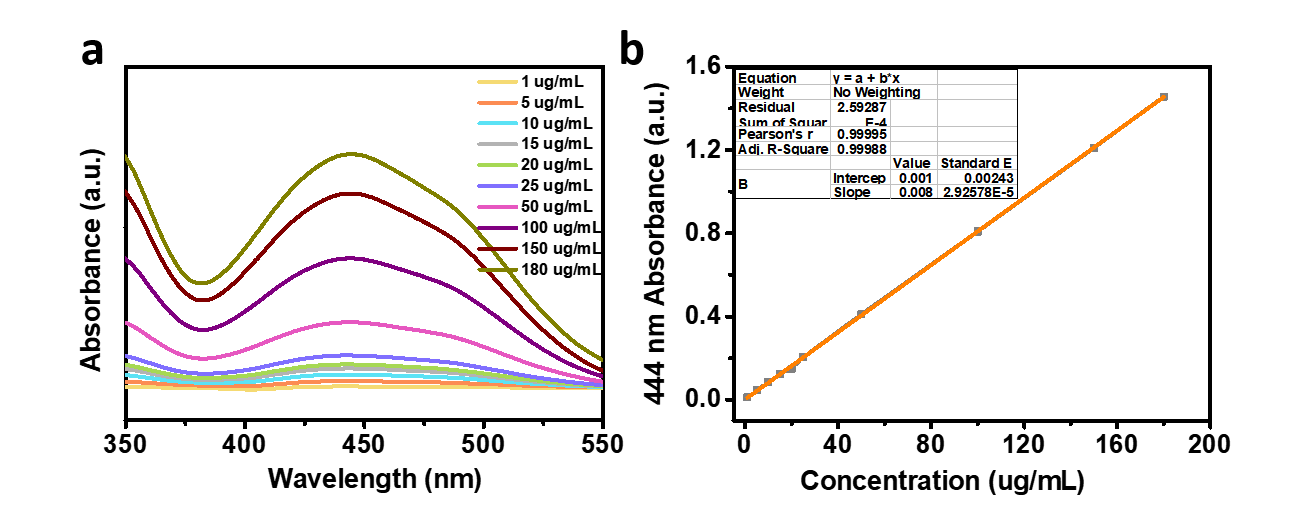


Fig. S7. (a) UV–vis absorption spectra of the solutions containing different concentrations of Lapa and (b) its standard curve.


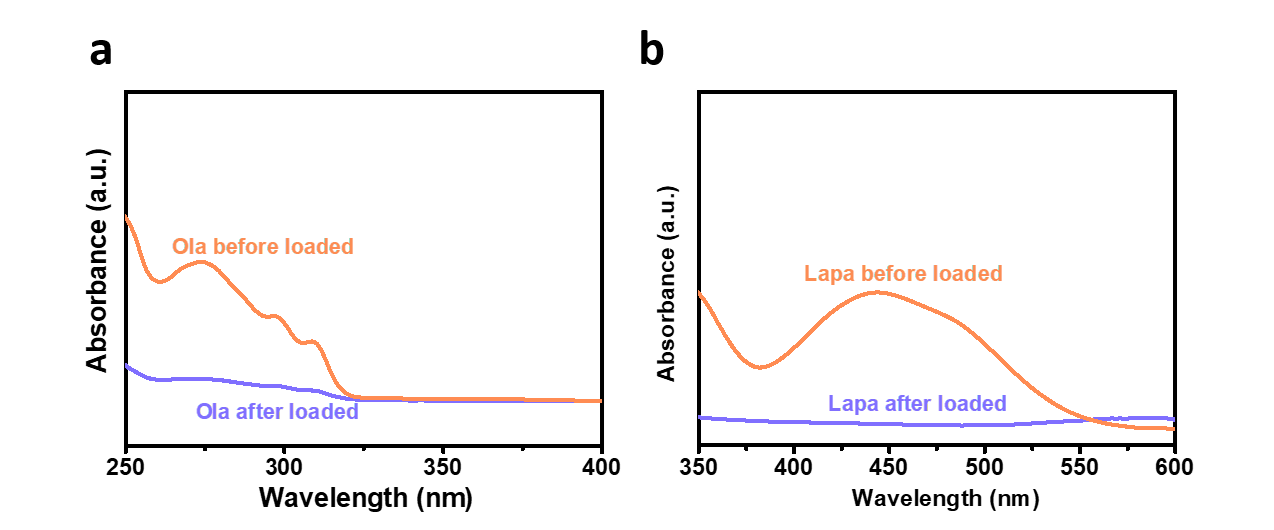


Fig. S8. UV-vis absorbance spectra of Ola, and Lapa before and after loading. The loading capacity was approximately (a) 14.5±0.45% for Ola, and (b) 31.1±0.48% for Lapa,.


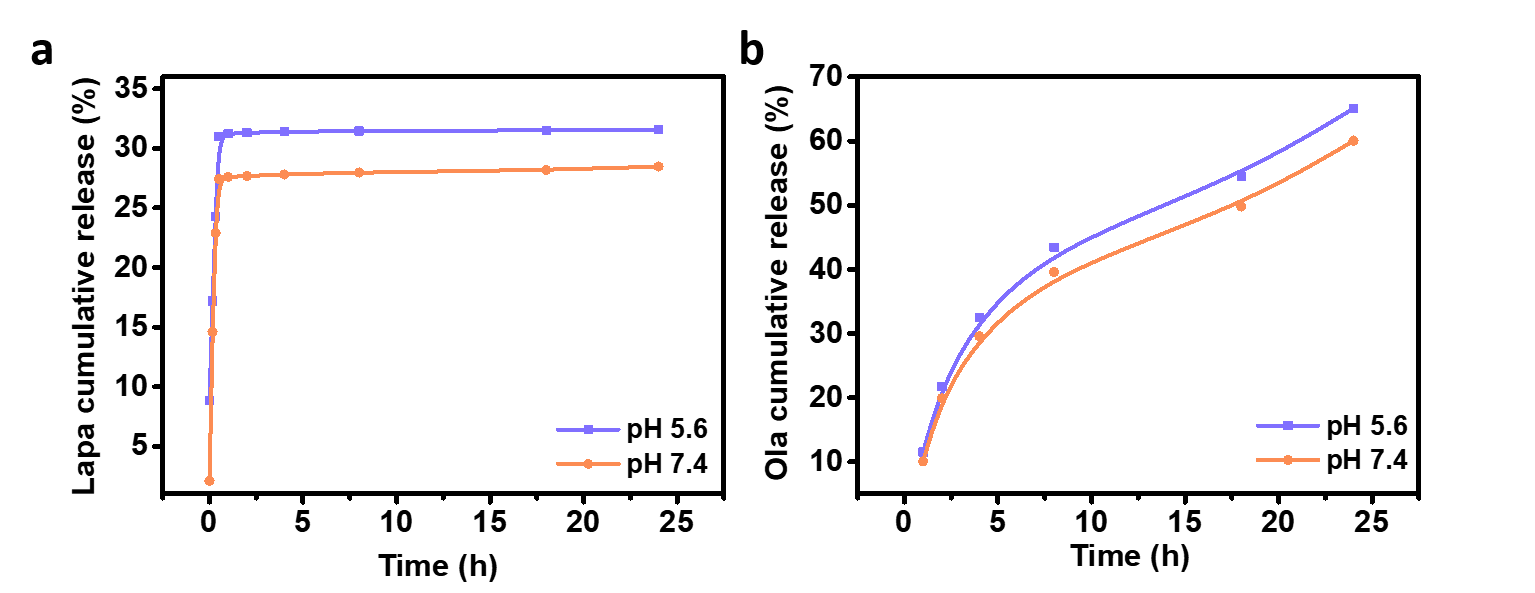


Fig. S9. Drug release behavior of (a) Lapa and (b) Ola at different pH.


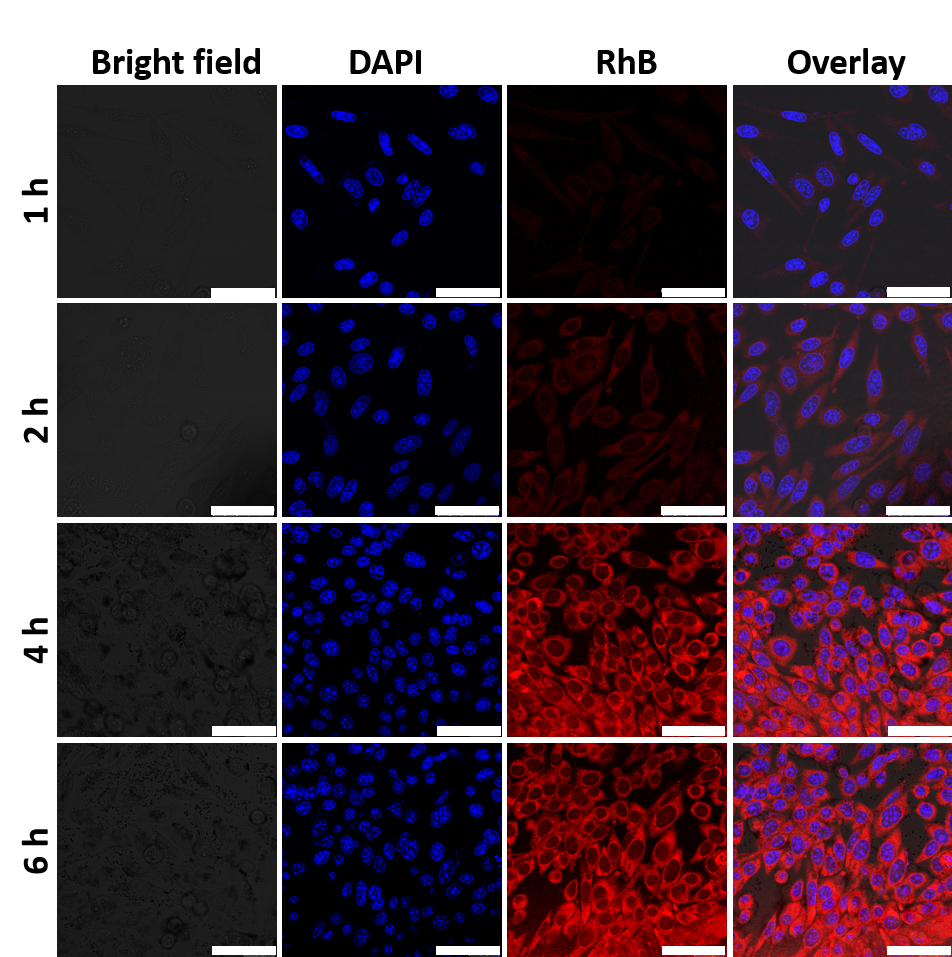


Fig. S10. CLSM images of MDA-MB-231 cells incubated with ZIF67/Ola/Lapa for different time periods at 5 µg/mL. Scale bar: 50 μm.


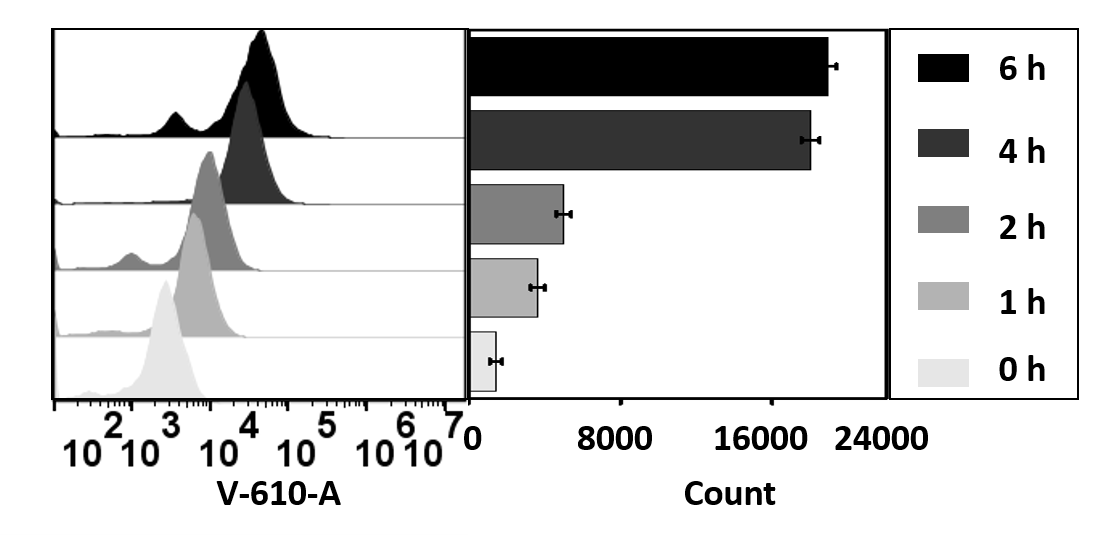


Fig. S11. Flow cytometry of MDA-MB-231 cells incubated with ZIF67/Ola/Lapa for different time periods at 5 µg/mL.


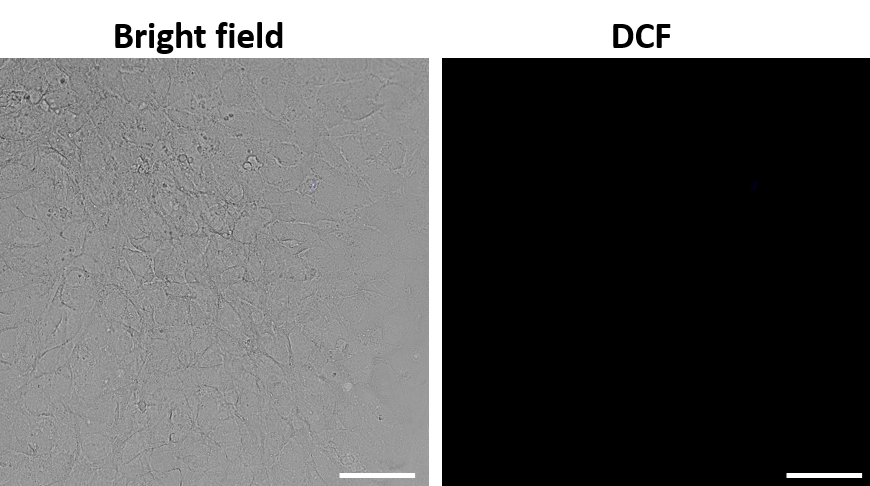


Fig. S12. CLSM images of intracellular ROS generation treated with ZIF67/Ola/Lapa in HBL-100 normal cells. Scale bar: 50 μm.


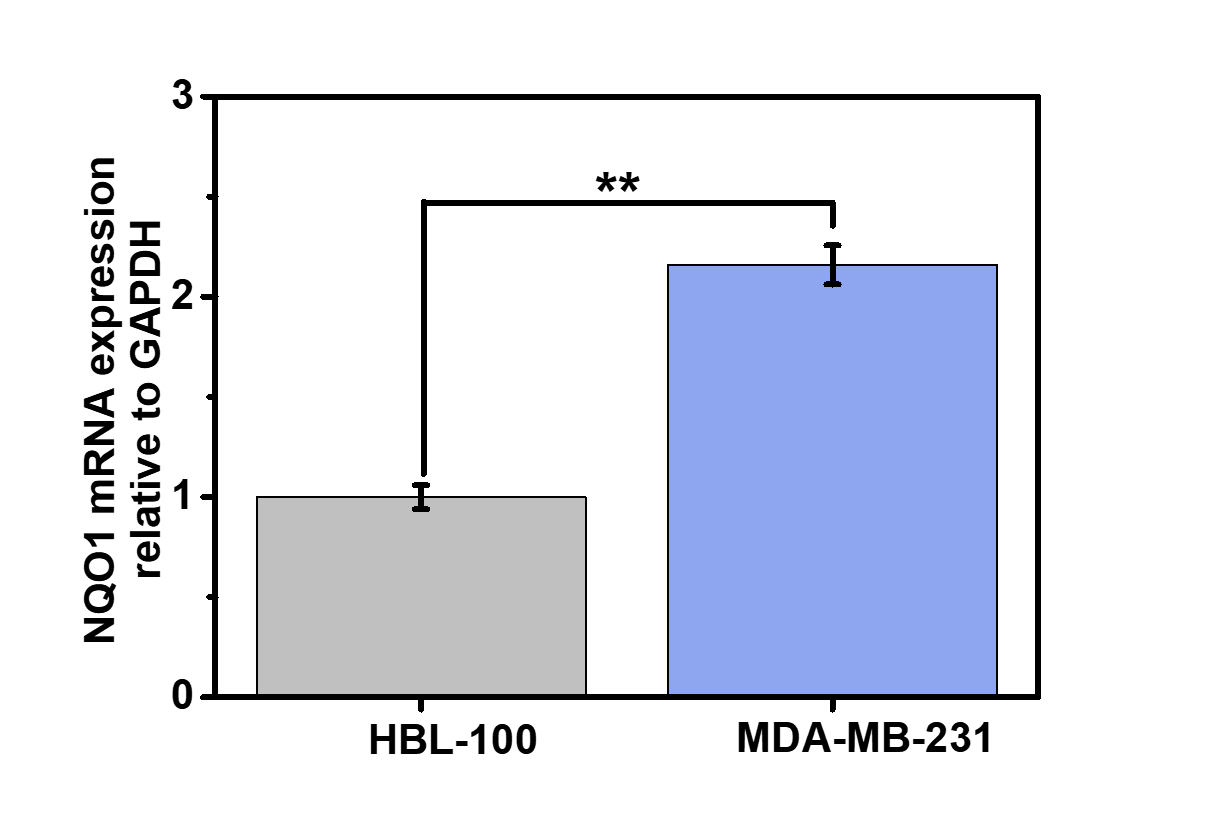


Fig. S13. PCR analysis of NQO1 expression level of HBL-100 normal cells and MDA-MB-231 breast cancer cells. (**p < 0.01)


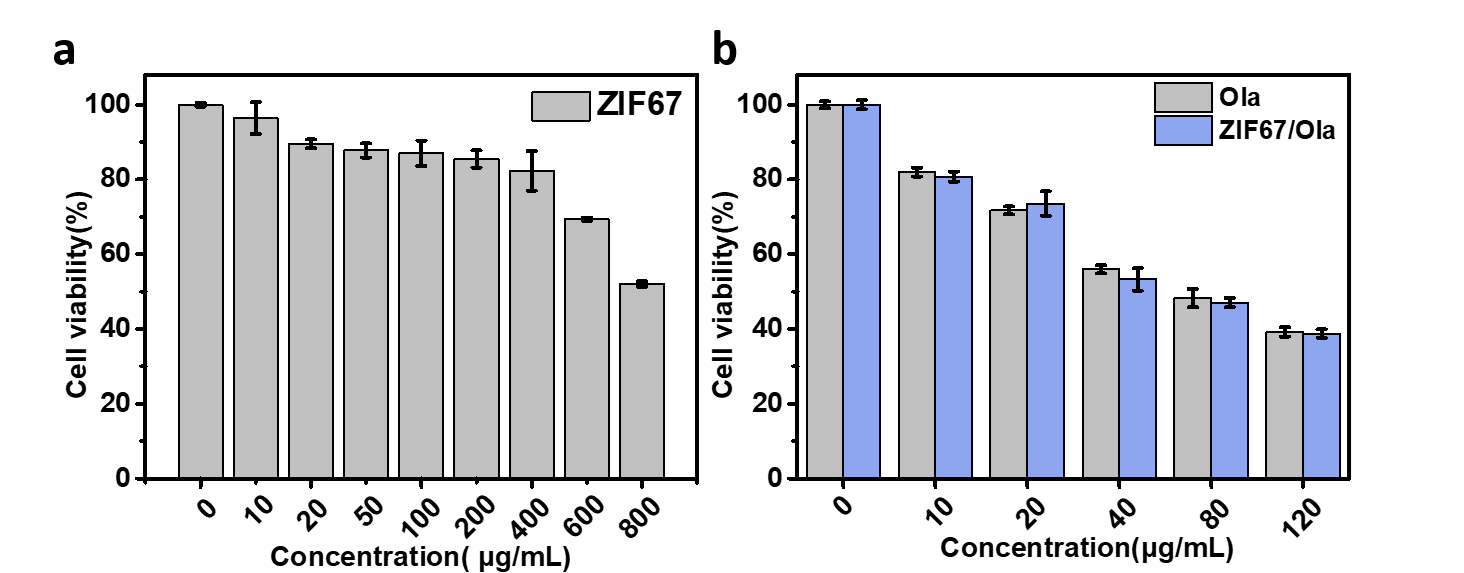


Fig. S14. The cytotoxicity of (a) ZIF67, (b) Ola and ZIF67/Ola towards MDA-MB-231 cells.


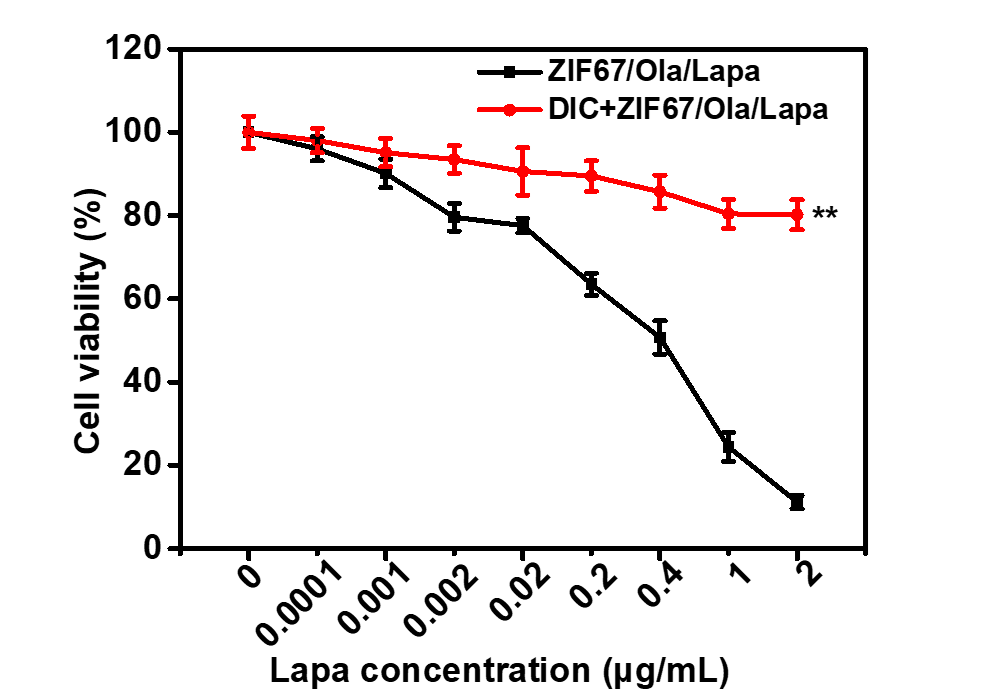


Fig. S15. The cytotoxicity of ZIF67/Ola/Lapa towards A549 cells or A549 cells pretreated with Dicoumarol (DIC).


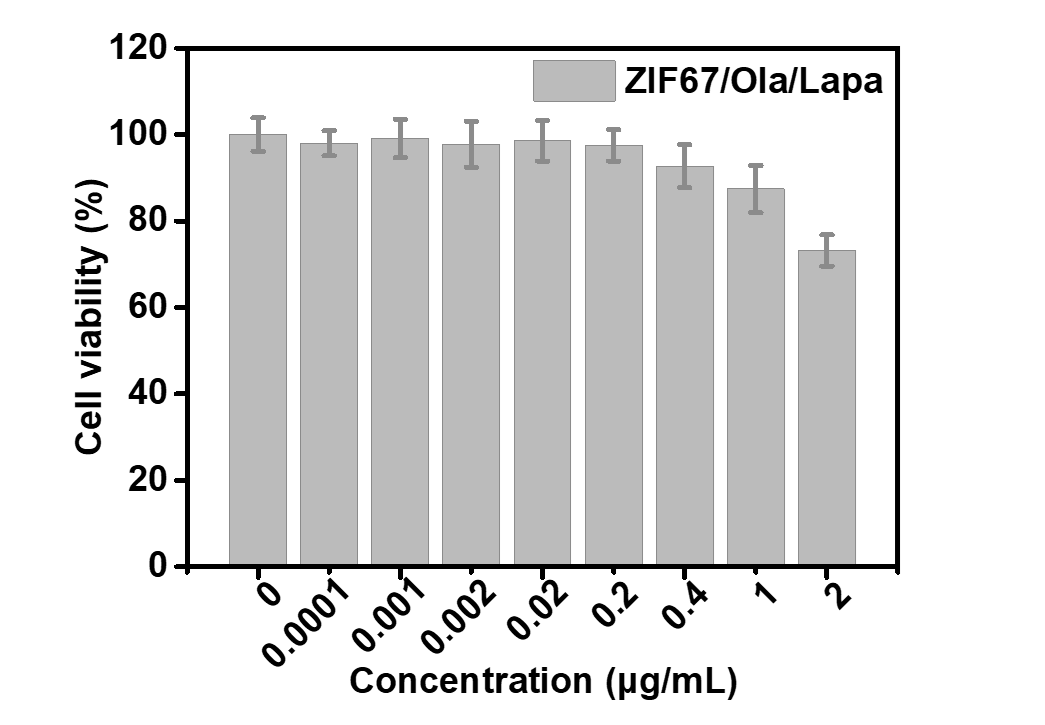


Fig. S16. The cytotoxicity of ZIF67/Ola/Lapa towards HBL-100 normal cells. *n=3*


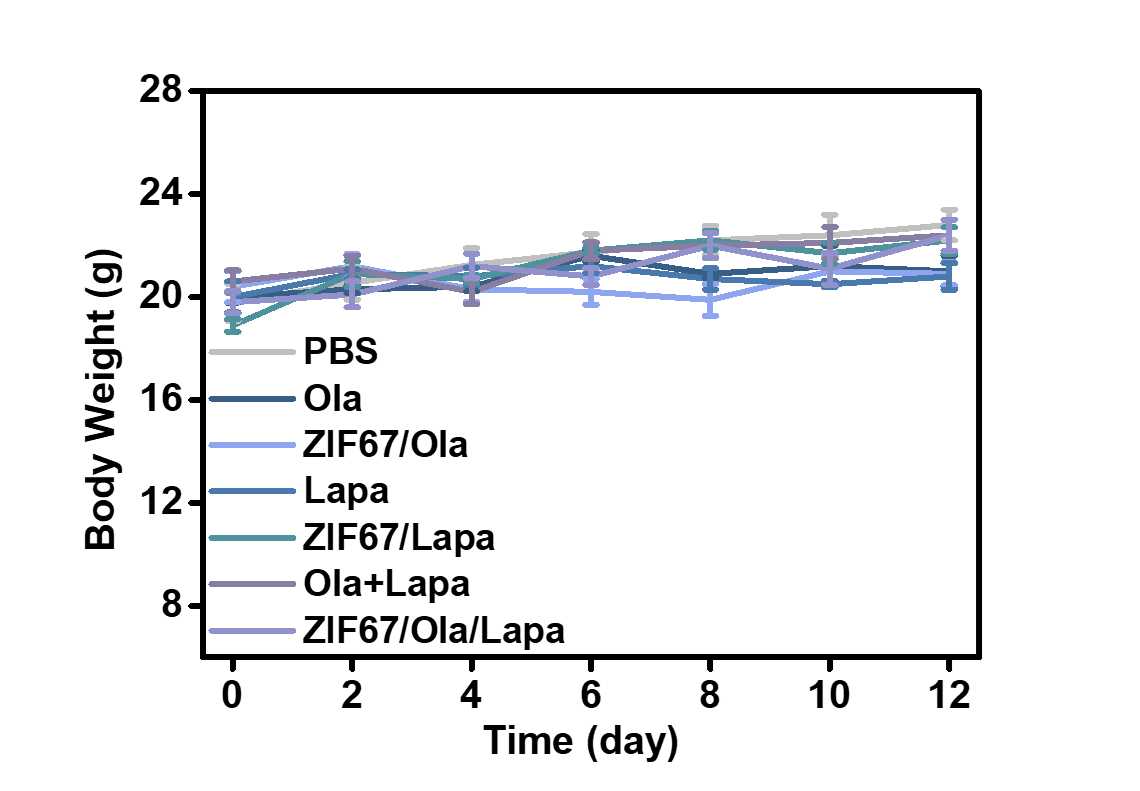


Fig. S17. Body weight of MDA-MB-231 tumor-bearing mice under different treatments.


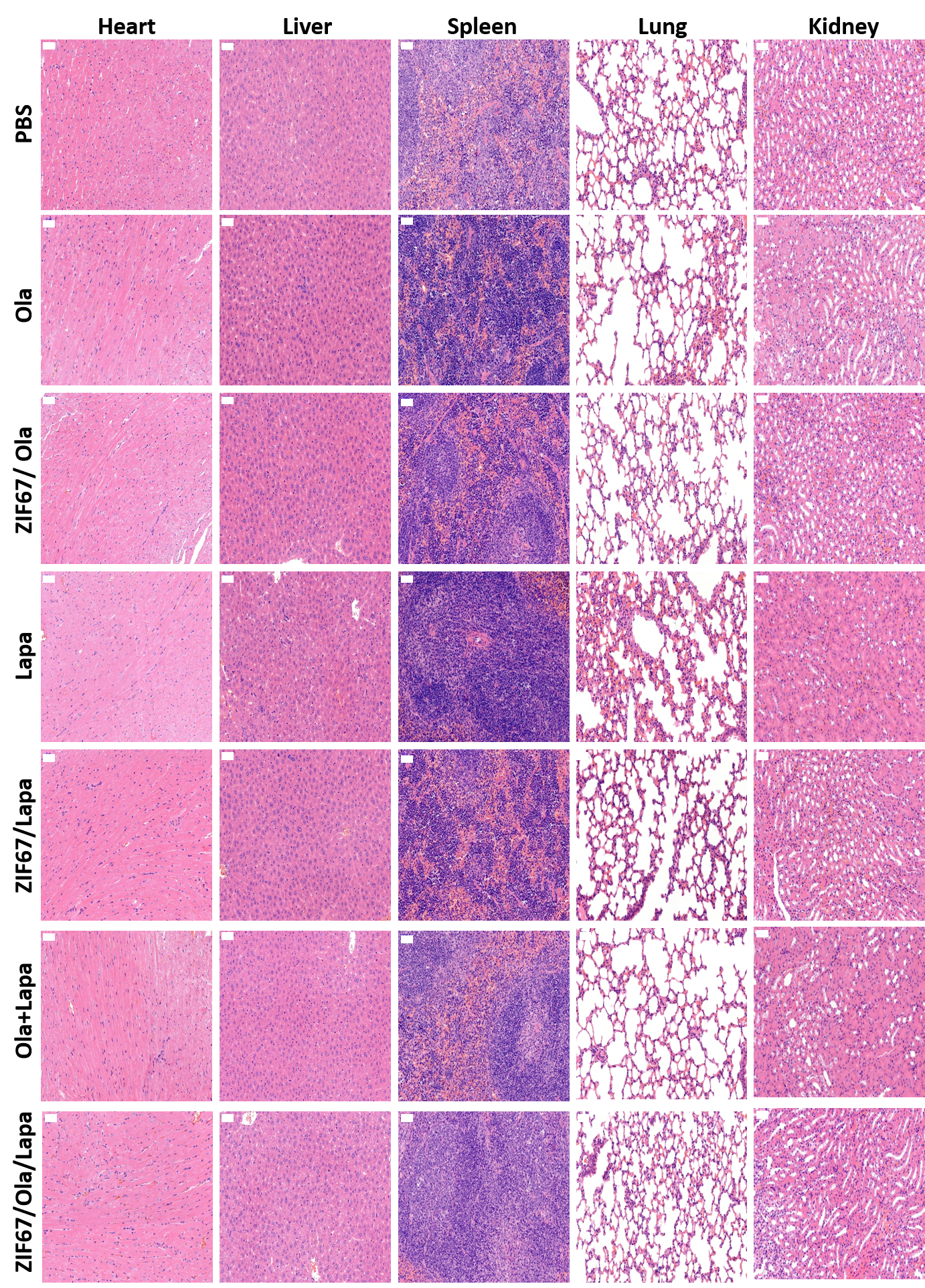


Fig. S18. H&E stained images of major organs obtained from different groups. Scale bar: 50 μm.


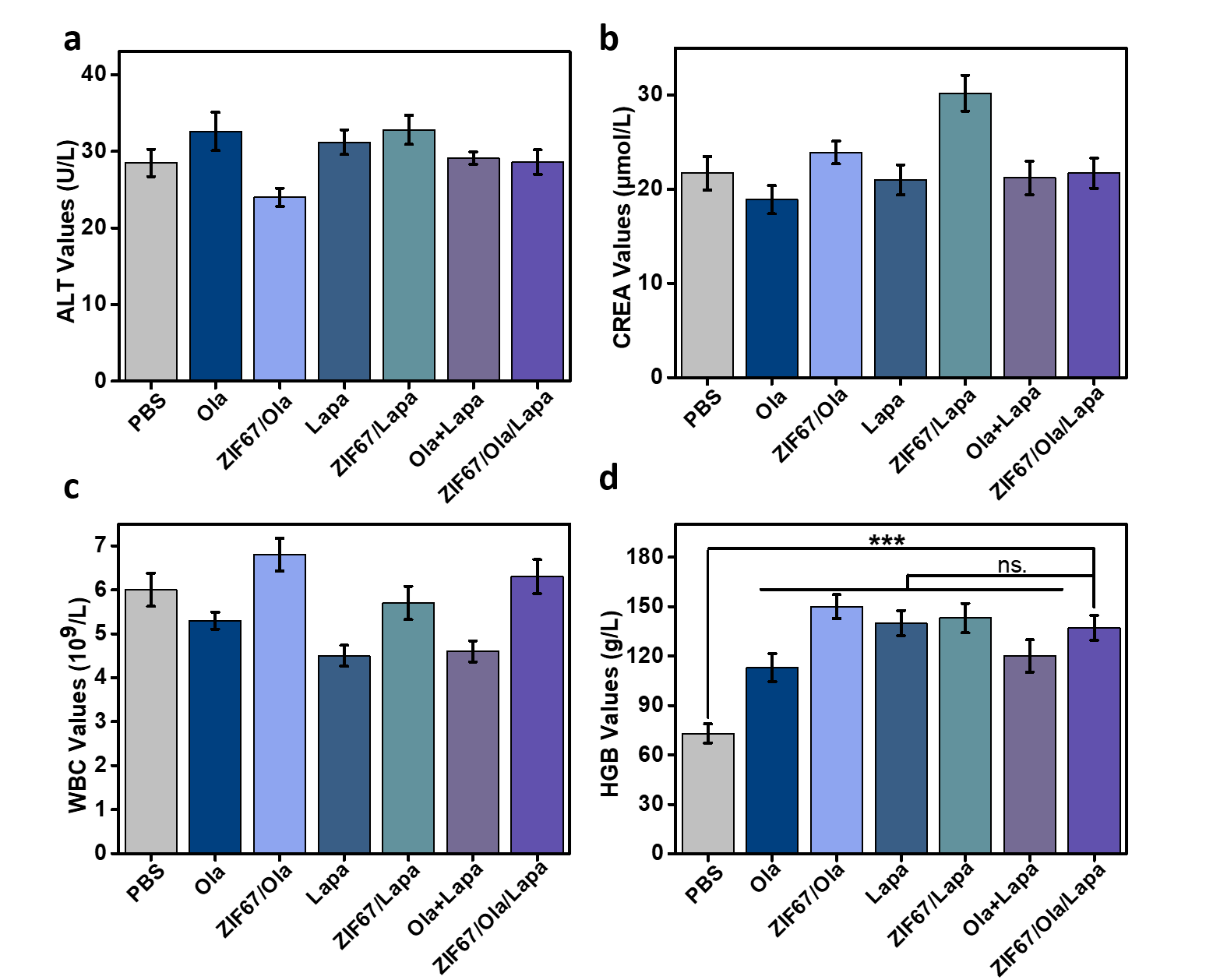


Fig. S19. Blood biochemistry data including numbers of (a) ALT, (b) CREA, (c) WBC, and (d) HGB. Data are shown as mean ± SD.

Table S1 The detailed data of drug loading capacity.

| **Drug** | **Initial fed weight (mg)** | **Weight of the supernatant (mg)** | **Weight of ZIF67/Ola/Lapa nanoparticles (mg)** | **Drug loading capacity (%)** | **Mean value of loading capacity (%)** |
| --- | --- | --- | --- | --- | --- |
| Ola-1 | 1.2 | 0.388 | 5.5 | 14.8 | 14.5±0.45 |
| Ola-2 | 1.2 | 0.351 | 5.7 | 14.9 |  |
| Ola-3 | 1.1 | 0.335 | 5.5 | 13.9 |  |
| Lapa-1 | 1.8 | 0.0772 | 5.5 | 31.3 | 31.1±0.48 |
| Lapa-2 | 1.9 | 0.169 | 5.7 | 30.4 |  |
| Lapa-3 | 1.8 | 0.0653 | 5.5 | 31.5 |  |
